# Identification of copy number variants contributing to hallux valgus

**DOI:** 10.3389/fgene.2023.1116284

**Published:** 2023-03-23

**Authors:** Wentao Zhou, Jun Jia, Hui-Qi Qu, Feier Ma, Junyi Li, Xiaohui Qi, Xinyi Meng, Zhiyong Ding, Gang Zheng, Hakon Hakonarson, Xiantie Zeng, Jin Li, Qianghua Xia

**Affiliations:** ^1^ Department of Cell Biology, The Province and Ministry Co-sponsored Collaborative Innovation Center for Medical Epigenetics, Key Laboratory of Immune Microenvironment and Disease (Ministry of Education), School of Basic Medical Sciences, Tianjin Medical University, Tianjin, China; ^2^ Department of Surgery of Foot and Ankle, Tianjin Hospital, Tianjin, China; ^3^ Center for Applied Genomics, Children’s Hospital of Philadelphia, Philadelphia, PA, United States; ^4^ Mills Institute for Personalized Cancer Care, Fynn Biotechnologies Ltd., Jinan, China; ^5^ National Supercomputer Center in Tianjin (NSCC-TJ), Tianjin, China; ^6^ Division of Human Genetics, Children’s Hospital of Philadelphia, Philadelphia, PA, United States; ^7^ Department of Pediatrics, Perelman School of Medicine, University of Pennsylvania, Philadelphia, PA, United States; ^8^ Department of Bioinformatics, School of Basic Medical Sciences, Tianjin Medical University, Tianjin, China

**Keywords:** hallux valgus, whole exome sequencing, copy number variation, immune dysregulation, cytochrome p450 metabolism

## Abstract

Hallux valgus is a common form of foot deformity, and genetic factors contribute substantially to the pathogenesis of hallux valgus deformity. We conducted a genetic study on the structural variants underlying familial hallux valgus using whole exome sequencing approach. Twenty individuals from five hallux valgus families and two sporadic cases were included in this study. A total of 372 copy number variations were found and passed quality control filtering. Among them, 43 were only present in cases but not in controls or healthy individuals in the database of genomic variants. The genes covered by these copy number variations were enriched in gene sets related to immune signaling pathway, and cytochrome P450 metabolism. The hereditary CNVs demonstrate a dominant inheritance pattern. Two candidate pathogenic CNVs were further validated by quantitative-PCR. This study suggests that hallux valgus is a degenerative joint disease involving the dysregulation of immune and metabolism signaling pathways.

## Introduction

Hallux valgus (HV) deformity refers to the lateral deflection of the great toe at the first metatarsophalangeal joint (Perera et al., 2011; Hecht and Lin, 2014), which is the most common forefoot deformity often requires surgery with a prevalence rate of 23% in people aged 18–65 (CI: 16.3%–29.6%) (Nix et al., 2010). African Americans are more likely to have HV than people of European ancestry [adjusted odds ratio (aOR) = 2.01, 95% confidence interval [CI] = 1.39–2.92] (Golightly et al., 2012), and no significant difference has been found in the incidence of HV in other populations. Conservative treatment is feasible for patients with deformity but without symptoms or mild symptoms (Bayar et al., 2011). Although the symptoms can be alleviated, they can not be completely reversed. If the patient’s pain persists, surgery is necessary. However, many surgical complications and poor prognosis have brought great burden to individuals and their families (Sammarco and Idusuyi, 2001). Prevention and early intervention are therefore important.

The pathogenesis of HV deformity is complex (Mann and Coughlin, 1981). HV may be associated with inappropriate footwear. HV is 15 times more common in people who wear shoes than in those who don’t (Perera et al., 2011), and shoes that tighten the front foot appear to be one of the leading causes of HV. Heredity is another important risk factor for HV development, especially in adolescents. A positive family history of HV has been reported in many studies (Lieberson and Mendes, 1991; Pique-Vidal et al., 2007), and study has found that HV susceptibility is related to genetic polymorphisms associated with arthritis (Perera et al., 2011; Hecht and Lin, 2014). Linear bone arrangement or static stabilizer relaxation due to heredity may also lead to HV deformity (Perera et al., 2011).

Genetic factors make substantial contribution to the pathogenesis of HV deformity. In a study of 350 patients with three generations family trees, 90% had at least one affected relative, which is consistent with autosomal dominant inheritance pattern (Pique-Vidal et al., 2007). The heritability of HV in European ancestry populations is between 0.29 and 0.89 (Hannan et al., 2013), while the rate of HV in Korea is ∼0.51 (Lee et al., 2014). In a genome-wide association study (GWAS) on European ancestry population including 1786 cases of HV deformity and 2623 controls, genome-wide SNPs accounted for 50% of the phenotypic variance in males and 48% of the phenotypic variance in females (Hsu et al., 2015). The missing heritability may lie in the contribution of rare variants and structural variants, which have been underexplored in previous studies.

Structural variation is generally defined as a region of DNA of approximately 1 kb or larger that includes changes in copy number, chromosomal position, or orientation between individuals (Escaramis et al., 2015). A major class of genomic structural variation is copy number variation (CNV), which includes deletion and duplication of sequences (Ionita-Laza et al., 2009). In addition to single nucleotide polymorphisms (SNP) (Sachidanandam et al., 2001), CNVs are a major source of variation in the human genome, with significant effects on evolution and disease susceptibility (Conrad and Hurles, 2007). For example, at least 15% of neurodevelopmental diseases are caused by local dose imbalances in dozens of genes due to CNVs (Girirajan et al., 2011). Furthermore, CNVs make significant contributions to development of bone disorders and CNV analysis increases the diagnostic yield for these diseases. CNVs are significantly associated with osteoporosis (Yang et al., 2008; Costantini et al., 2018) and CNV is an important genetic factor for the etiology of fetal skeletal dysplasia (Wit et al., 2014; Bai et al., 2022). In addition, studies have shown that many CNVs confer greater disease risk than SNPs (Ionita-Laza et al., 2009). However, the contribution of CNVs to the pathogenesis and development of HV has not been investigated.

Aiming to explore the contribution of potential pathogenic CNVs to the development of HV deformity, we conducted the first study on the structural variants underlying familial HV using whole exome sequencing (WES) approach, which may be helpful for future risk prediction of HV.

## Materials and methods

### Samples and ethics statement

**FIGURE 1:**
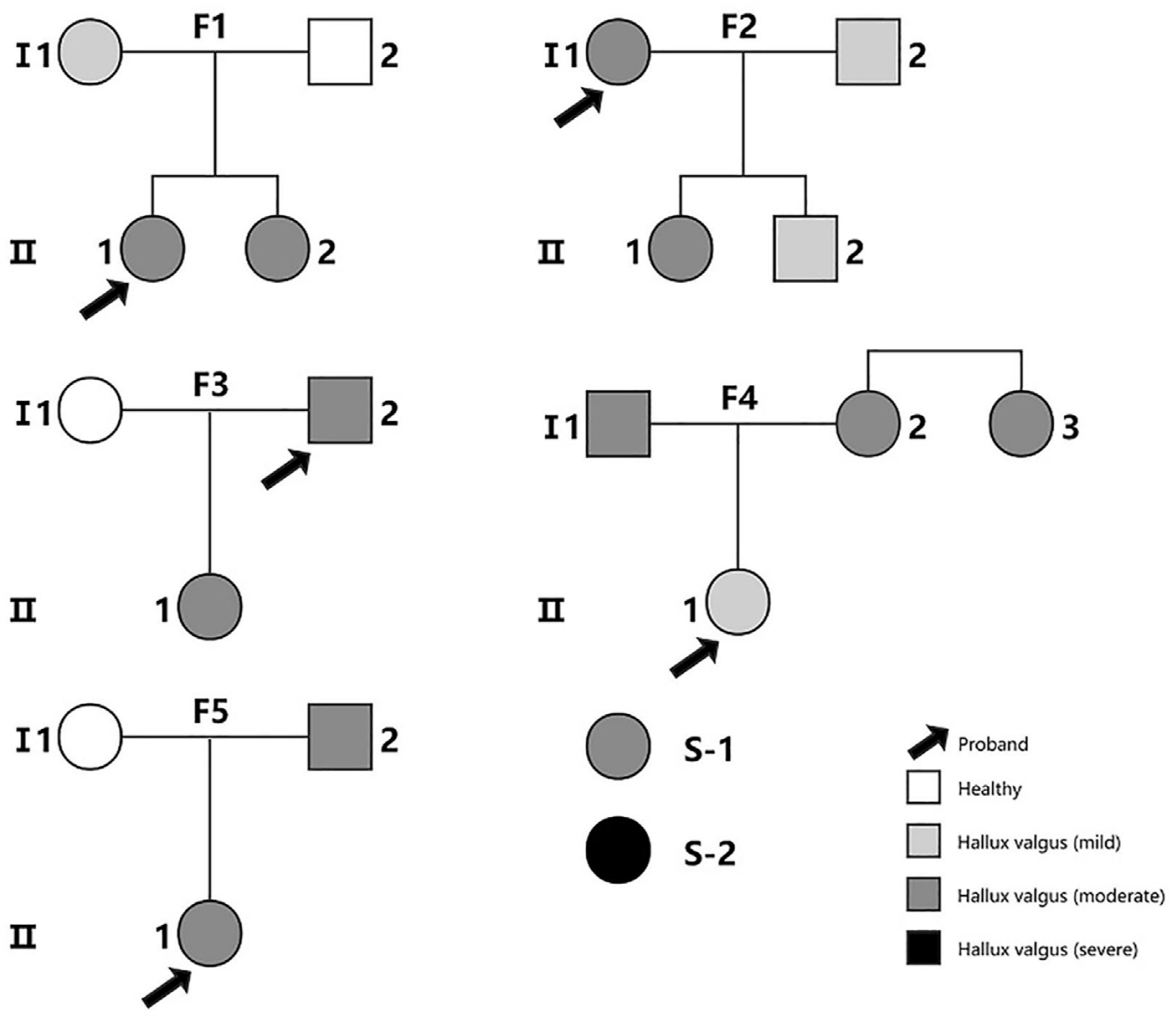
The family pedigree diagrams for all the subjects in the study. S-1 and S-2 are two sporadic cases. Families F1, F3 and F5 were included in our previous study of single nucleotide variants (Jia et al., 2021).The color indicates the severity of the patient’s illness from mild to severe.

In this study, we recruited a total of 22 Chinese participants from 5 families (including 17 cases and 3 controls without any foot deformity) and two sporadic cases (Figure 1). The recruitment and the WES study were approved by Tianjin Hospital, and all participants provided written informed consent. The diagnosis of HV was made by clinical experts of the Foot and Ankle Surgery Group of Orthopaedic Branch of Chinese Medical Association and Foot and Ankle Surgery Professional Committee of Orthopaedic Physician Branch of Chinese Medical Association, according to the expert consensus (Foot and Ankle Working Committee et al., 2015). HV is diagnosed by combining the evaluation of clinical presentations, physical examination, auxiliary imaging examination and medical history. The severity of HV was determined by hallux valgus angle (HVA) and intermetatarsal angle (IMA) (normal: HVA < 16°, IMA < 10°; mild: HVA < 20°, IMA < 13°; moderate: 20° < HVA ≤ 40°, 13° < IMA ≤ 16°; severe: HVA > 40°,IMA > 16°). The single nucleotide variant analysis of the WES data of three families has been reported in our previous publication (Jia et al., 2021). With the addition of data from HV families and sporadic cases, we performed the current CNV study. The genetic genealogy of the 5 families shows that each proband has at least one first-degree relative as a HV patient.

### Genomic DNA extraction and whole-exome sequencing

Genomic DNA was extracted from peripheral blood sample of each subject following standard procedures. The TargetSeqTM Enrichment Kit (iGeneTechTM) Human Exome Capture Kit was used for library construction. The Illumina sequencing platform was used for paired next-generation sequencing.

### Quality control of sequencing result files

The Trim Galore software (Martin, 2011) (https://github.com/FelixKrueger/TrimGalore) was used to remove low-quality base (−q 25), limit maximum allowable error rate (default −e 0.1), remove reads < 36 nt (-length 36), remove double-ended overlap>3, and remove reads as a unit (-paired) from the raw fastQ files. Burrows-Wheeler Aligner (BWA)-MEM (version 0.7.17) (Li, 2013) was used to align the reads with the reference genome to obtain the SAM file.

After reordering SAM files were converted to BAM (Raw BAM) files and polymerase chain reaction (PCR) duplicates were marked using Picard (v1.91). SamTools (version 1.58) (Li et al., 2009) was used for quality control of the binary alignment graph files generated between them. Then, the Genome Analysis ToolKit (GATK) (version 3.8) (McKenna et al., 2010) was used to re-align indel regions and correct the base mass fraction. And after Base quality score recalibrations (BQSRs), we used verifyBamID (version 1.1.3) (Jun et al., 2012) to confirm that there was no contamination of the cross samples. The resulting BAM files were used for CNV calling.

### CNVs calling by GATK and XHMM

**FIGURE 2:**
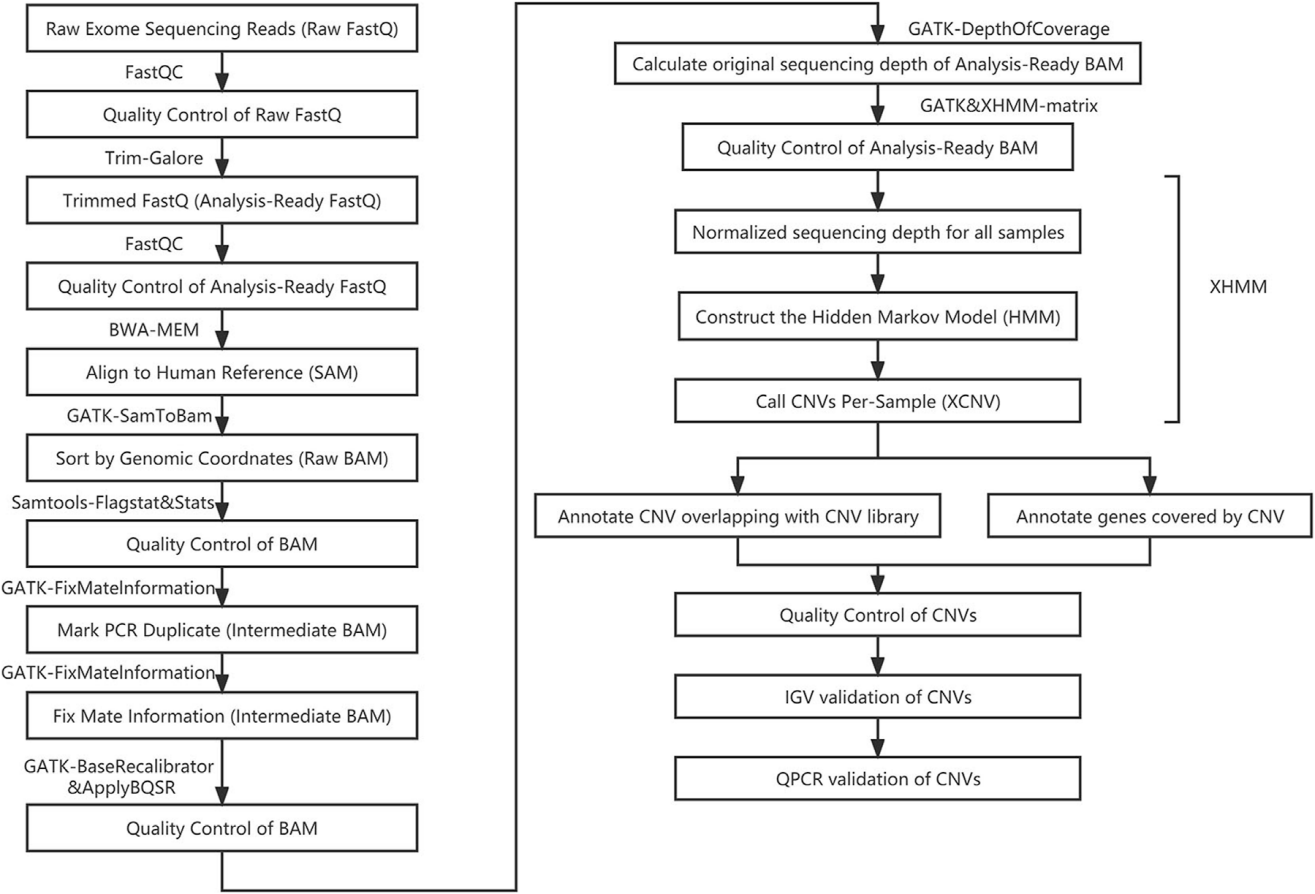
The pipeline of copy number variation analysis based on whole exome sequencing data.

The eXome-Hidden Markov Model (XHMM) (Fromer and Purcell, 2014) was used to call CNVs from filtered normalized targeted exome sequence data. The XHMM divided the chromosomal regions into three types: diploid, deletion, and duplication. The entire pipeline is plotted as a flowchart and shown in Figure 2.

### CNVs annotation

We used “scan_regoin.pl” program in GenGen(Wang et al., 2007) to scan genomic features and to find the CNVs that overlapped with those in the database of genomic variants (DGV) (MacDonald et al., 2014) and filtered them out. The DGV database contains genomic structural variants over 50 bp in healthy individuals.

We used “scan_regoin.pl” program in GenGen (Wang et al., 2007) with the hg19_refGene and hg19_refLink files to annotate the CNV regions against the RefGene annotation to find the genes overlapped the CNV regions.

Then, we filtered out CNVs present among controls, and retained case-only CNVs that were repeated among the HV cases but not carried by any of the controls.

### Quality control filtering of CNVs

We performed quality control filtering based on “Q_EXACT” and “Q_SOME” scores in the XCNV file according to the instruction of XHMM software. “Q_EXACT” and “Q_SOME” represent the phred-scaled quality of a CNV event along the entire interval and the same CNV event in the interval respectively.

### Pathway enrichment of CNVs

The 67 genes overlapped with the 43 CNVs were used as input to the web-portal of OBAS (http://kobas.cbi.pku.edu.cn/) (Bu et al., 2021) and STRING (version 11.0) (https://string-db.org/) (Szklarczyk et al., 2021) for pathway enrichment and protein-protein interaction (PPI) analysis. Kyoto Encyclopedia of Genes and Genomes (KEGG) pathway database and Search Tool for the Retrieval of Interacting Genes/Proteins database were used as the reference databases.

### Examination of CNVs using integrative genomics viewer

For candidate CNVs of interest, we further conducted visual inspection using the Integrative Genomics Viewer, based on the aligned bam files of the WES data of each CNV carrier and non-carriers within the same family.

### qPCR validation of CNVs

For the potential pathogenic CNVs, we chose a ∼100 bp fragment in each CNV for qPCR validation. The qPCR experiment was carried out with the Sybr Green I system. Each reaction in a 10 µL system contains 4 ng of genomic DNA, 10 µm per-primer, 2× ChamQ universal SYBR qPCR Master Mix, and DD-H_2_O. Each sample was repeated for three times. The geometric mean values of the CT values of the control sequence GAPDH and the sample sequence were calculated to obtain △CT values for each sample. We calculated the 2^−△△CT of each sample by taking the 2^−△ CT of members without CNV in the family as the relative reference value. Finally, the existence of CNV was examined by the relative value of 2^−△△CT of each sample in the pedigree.

## Results

### The identification of 43 CNVs related to HV

To assess the potential contribution of structural variants to HV, we carried out a CNV study based on the WES data of five HV families including 17 cases and 3 family members without HV and two sporadic cases (Supplementary Table S1). A total of 965 CNVs were detected based on the WES data and 372 passed quality control filtering (Supplementary Table S2).

Among these 372 CNVs, 325 CNVs were present only in cases, including 142 deletions and 183 duplications. They were not detected in the any of the three control samples. Furthermore, 51 CNVs (26 deletions and 25 duplications) were carried by more than one samples. In order to screen for potential pathogenic CNVs, we further filtered out any CNVs with 70% overlap with those carried by healthy individuals in the DGV. Then, 43 CNVs were retained for further analysis, covering the exons of the 67 genes, with 1 CNV co-occurring in four cases, 5 CNVs co-occurring in three cases, and 37 CNVs co-occurring in two cases (Supplementary Table S3).

### The involvement of immune and metabolism genes

**FIGURE 3:**
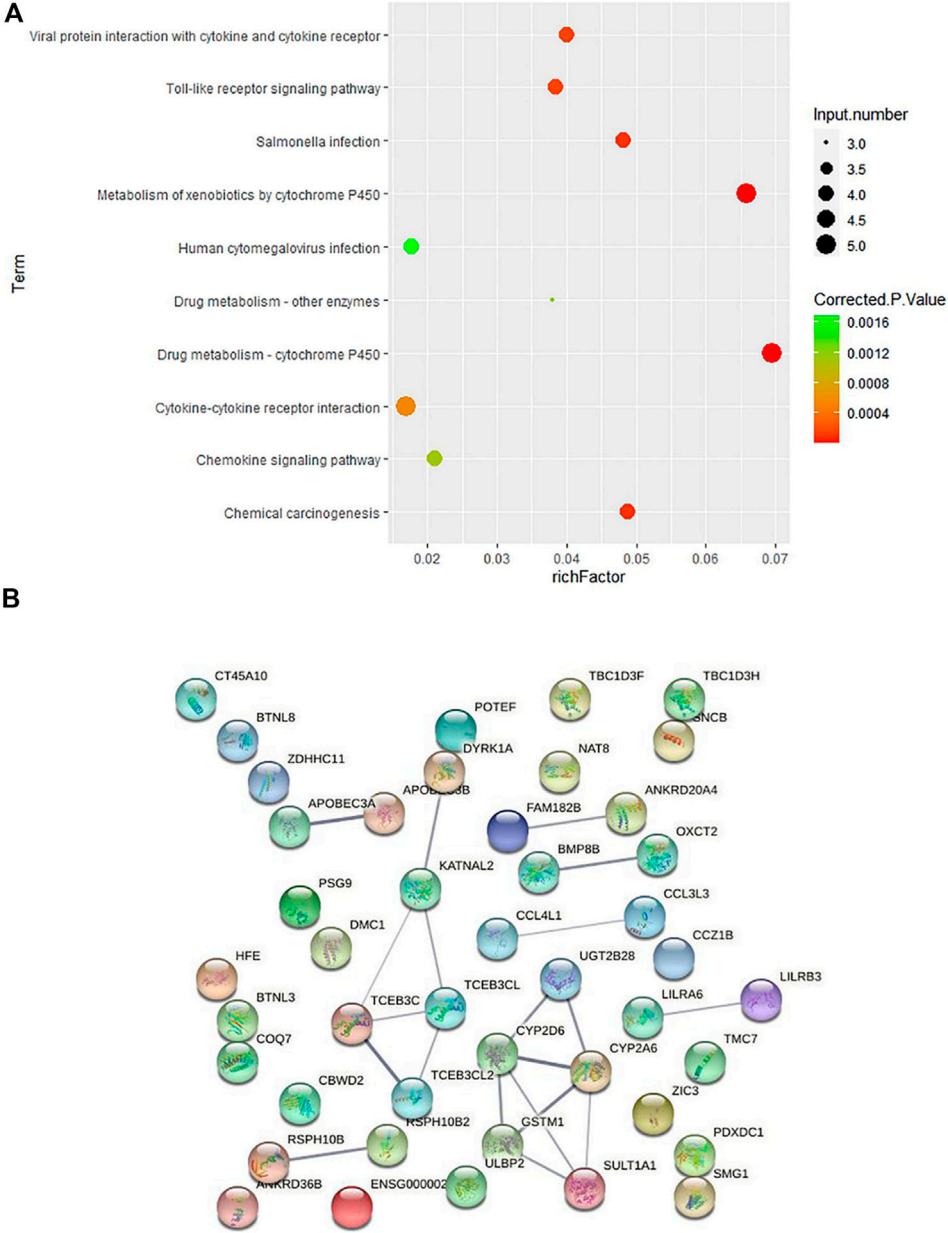
Pathway and protein-protein interaction analyses on the genes covered by 43 case-only CNVs **(A)** KEGG pathway enrichment analysis on the genes covered by the 43 case-only CNVs. The color of the dots represents the size of the corrected *p*-value, and the size of the dots represents the number of input genes contained in the corresponding pathway. **(B)** Protein-protein interaction analysis on the genes covered by 43 case-only CNVs. The nodes represent the proteins, and the lines between the nodes indicate the interactions between the proteins.

In order to further explore how these CNVs may contribute to the pathogenesis of HV, we carried out pathway enrichment analysis among the 67 genes covered by case-only CNVs through KEGG PATHWAY database. Ten pathways were statistically significantly enriched (*p* < 0.001) for cytokine genes and genes involved in cytochrome P450 related metabolism and immunity/inflammation (Figure 3A; Supplementary Table S4), such as KEGG pathways “Drug metabolism—cytochrome P450”, “Toll-like receptor signaling pathway” and “Cytokine-cytokine receptor interaction”, suggesting their contribution to the pathogenesis of HV.

We then conducted the PPI analysis on these genes (Figure 3B). The PPI network also supports the consistent involvement of a group of immune genes and genes functioning in drug metabolism. In the PPI network, we observed the cluster formed by proteins involved in drug metabolism (UGT2B28, CYP2D6, CYP2A6, GSTM1, SULT1A1). In addition to the well-known involvement of cytokine genes CCL4L1 and CCL3L3 in immunity, several other genes also function in regulation of the immune system. HLA-H (HFE) is an important link between iron homeostasis and immune regulation (Bahram et al., 1999; Lio et al., 2002); DYRK1A gene plays a key role in regulating the differentiation of Th17 and regulatory T cells (Khor et al., 2015); and evidence suggests that genetic polymorphisms of CYP2D6 is associated with autoimmune bullous diseases induction (Rychlik-Sych et al., 2013).

### Inheritance pattern of HV-related CNVs

To identify the potential disease-contributing CNVs, we examined the inheritance pattern of the CNVs which passed quality control filtering. We did not find any CNV that fits the recessive pattern. Multiple case-only CNVs consistent with dominant inheritance pattern were found in each family. For family 1, 3 and 5, dominant CNVs were referred to those present in cases (child and parent with HV) but absent in controls (parent without HV). For family 2 and family 4 in which all family members were affected individuals with HV, the CNVs consistent with dominant inheritance were those carried by the child and one of the parents (Supplementary Table S5). Our results are consistent with the hypothesis that HV has a dominant inheritance (Pique-Vidal et al., 2007), but certainly we can not exclude the possibility of recessive inheritance model as a large proportion of the genome were not assayed in our analysis by WES.

### Validation of disease-contributing CNVs

Two inherited CNVs were of particular interest and they have the highest quality (Phred-scaled quality of Non-Diploidy = 99) in our analyses (Table 1). Chr22: 42522498–42536739 deletion occurs to all patients in family 1 and absent in the healthy member of the family. This segment of CNV covered genes CYP2D7 and CYP2D6 which are involved in bone excitation effects (Jayaraman et al., 2021) and metabolic pathways related to human immunity (Effner et al., 2017). Chr6: 29855550–29895036 deletion appears to F2-I-1 and F2-II-1 the two individuals with moderate HV but not in family members F2-I-2 and F2-II-2 who have only mild HV condition. HCG4B and HLA-H genes are in this segment of CNV. HCG4B is a proinflammatory gene whose expression is positively correlated with the expression of HLA-A and may regulate the expression of HLA-A (Chen et al., 2017). HLA-A and HLA-H function in immune homeostasis (Jordier et al., 2019). Therefore evidence from literature suggests that these two CNVs may contribute to the pathogenesis of HV.

**TABLE 1:** Candidate pathogenic CNVs.

SAMPLE	CNV	INTERVAL	Q_NON_DIPLOID	Q_SOME	GENE
F1-I-1,F1-II-1,F1-II-2	DEL	chr22: 42522498–42536739	99	99	CYP2D6, CYP2D7
F2-I-1,F2-II-1	DEL	chr6: 29855550–29895036	99	93	HCG4B, HLA-H

SAMPLE, sample ID; CNV, type of copy number variation (DEL or DUP); INTERVAL, genomic range of the called CNV; Q_NON_DIPLOID, Phred-scaled quality of not being diploid, i.e., DEL or DUP event in the interval; Q_SOME, Phred-scaled quality of some CNV event in the interval; GENE, gene name.

**FIGURE 4:**
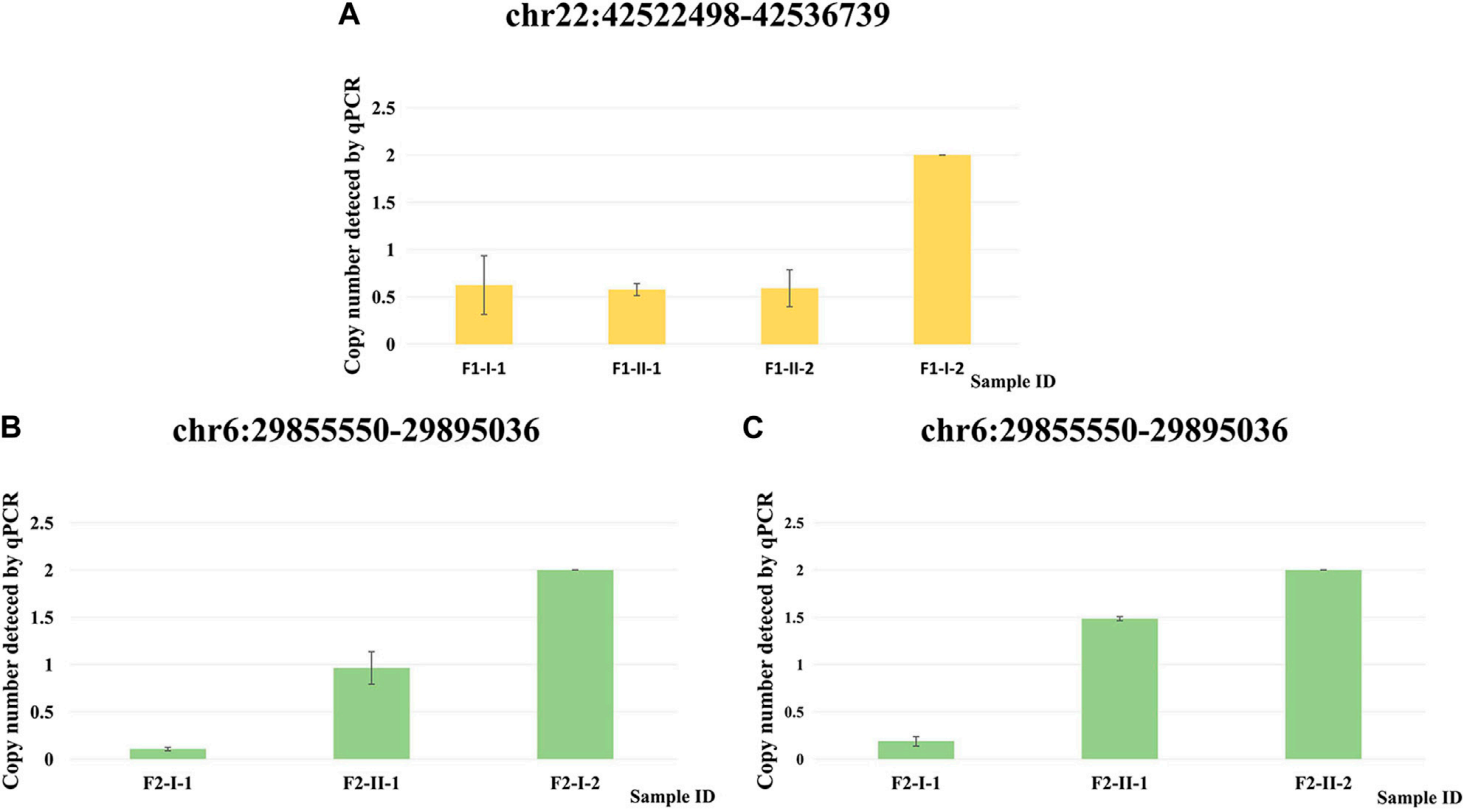
qPCR validation of two CNVs. **(A)** Copy number detected by qPCR at chr22: 42522498–42536739 for F1-I-1, F1-II-1, F1-II-2 and F1-I-2. **(B)** Copy number detected by qPCR at chr6: 29855550–29895036 for F2-I-1, F2-II-1 and F2-I-2. **(C)** Copy number detected by qPCR at chr6: 29855550–29895036 for F2-I-1, F2-II-1 and F2-II-2.

We performed qPCR (Supplementary Table S6) as an independent experimental approach to validate these two CNVs. The presence of these two CNVs in family 1 and family 2 respectively was consistent with the results from the CNV calling of WES data (Figure 4).

## Discussion

This is the first study that identified the structural variants underlying familial HV using WES approach, The identification provided us new knowledge about the genetics basis of HV, which highlighted the potential contribution of immunity/inflammation, and cytochrome P450 metabolism to the etiology of HV.

Previous studies indicated that HV malformations are likely to be caused by a variety of contributing factors, including genetics, dorsiflexion of the first metatarsal, gastrocnemius equinus, abnormal foot mechanics, and joint hypermobility (Coughlin and Jones, 2007). Interestingly, patients with autoimmune arthritis conditions such as rheumatoid arthritis and psoriatic arthritis are more likely to develop HV malformations (Rojas-Villarraga et al., 2009; Hyslop et al., 2010).

The CNV chr6: 29855550–29895036 observed in both F2-I-1 and F2-II-1 covers the HCG4B and HLA-H genes. It has been found that HCG4B is a pro-inflammatory factor and may regulate the expression of HLA-A by acting on competing endogenous RNAs spongingmiR-122 and miR-1352 (Chen et al., 2017). HLA-A and HLA-H play essential roles in the immune system, including regulation of the innate immune system, class I MHC-mediated antigen processing and presentation, interferon gamma signaling, and antigen processing-cross-presentation (Allan et al., 2002; Nitschke et al., 2016; Jordier et al., 2019). In psoriatic arthritis and rheumatoid arthritis, HLA alleles have been shown to influence susceptibility and severity of these autoimmune joint disease (Mc et al., 2015).

The CNV chr 22: 42529569–42529669 seen in F1-I-1, F1-II-1 and F1-II-2 covers CYP2D6 and CYP2D7.These genes have roles in the cytochrome P450 related xenobiotic metabolic process and oxidation-reduction process (Lessard et al., 1997). Cytochrome P450 changes the structure of cell antigens by metabolizing foreign organisms to produce reactive oxygen species, and then initiates and/or amplifies the autoimmune phenomenon through molecular simulation of the autoimmune response to the original antigen (Namazi, 2009). In addition, studies have shown that secondary metabolites produced by CYP2D6-dependent biotransformation have bone excitation effects (Jayaraman et al., 2021).

These two CNVs may have pathological effects and lead to bone structure deformities through the dysregulation of the immune system and the process of reactive oxygen species production.

Previous genetic studies on HV, including our WES study (Jia et al., 2021), revealed the association of a few genetic variants with HV. Genome-wide association study in population of European ancestry found the sex-specific association of SNVs close to genes AXIN2, ESD, ANXA1 and MRGPRX3 at the marginal significance level (Hsu et al., 2015). A later GWAS with increased sample size identified the genome-wide significant association with SNP in gene CLCA2, being an expression quantitative trait locus for COL24A1 (Arbeeva et al., 2020). Candidate gene study in Chinese population suggested variants in VDR gene and SNP rs1800629 at the 5’ of TNF gene are related to HV (Tao et al., 2018; Arbeeva et al., 2020). In our study, we did not find outstanding CNVs in these genes, which could be due to differences in study population, variant types. We also focused on different regions of the human genome from the previous GWAS (the coding exons *versus* the non-coding regions). However, similar underlying genetic mechanisms were revealed. As discussed by Hsu and colleagues, AXIN2 and ANXA1 have important functions in regulation of innate and adaptive immunity, suggesting the potential role of inflammation in HV (Hsu et al., 2015). Hsu and colleagues also discussed the involvement of the serine hydrolase gene ESD gene in the recycling of sialic acids which is related to decreased antioxidant levels. Interestingly, CYP2D6 and CYP2D7 also have roles in oxidation-reduction process. The SNP rs1800629 associated with HV in Chinese population is an eQTL SNP for genes including complement gene C4A and CYP21A1P a member of the Cytochrome P450 Family. Therefore, these identifications and results from our study converge on the similar immune and drug metabolism signaling.

Because of the importance of CNV in the etiology of complex human diseases, various CNV detection methods and computational algorithms have been developed. The traditional array-based approach including array-based comparative hybridization and SNP-array approaches can efficiently detect large CNVs with a relatively accurate rate (Gabrielaite et al., 2021). CNV calling from high-throughput sequencing data has become more and more commonly utilized in research and clinical diagnosis of complex human disorders. Though still being limited by short read length, high-throughput sequencing offers the potential to identify novel CNVs in the genomic regions with sparse coverage or lack of coverage by array probes. As the most frequently used sequencing approach, WES focuses on the exomes, the copy number alterations of which are more likely to have a pathogenic effect than the other regions of the human genome. Calling CNVs from WES data also has the advantages of detecting small CNVs ranging from 1–100 kb (Gordeeva et al., 2021). This approach has the drawback due to non-uniform read depth distribution between exons which makes it prone to false positive detection (Ozden et al., 2022). It is also subjected to missing CNVs in regions with few exons. As another sequencing-based CNV detection approach, CNV-seq technology can potentially provide a unbiased coverage of the human genome and more precise estimation of CNV breakpoints (Liang et al., 2014), however it also has the limitation of relative low read depth and resolution of 0.1 Mb in size which render it more suitable to detect large CNVs. Owing to the low cost, popularity in research use, abundant existing data, simultaneous usage for single nucleotide variant detection, and a number of well-tested CNV-calling algorithms, WES data are still a common choice of CNV analysis. With the gradually reduced sequencing cost and further improved CNV-calling algorithms, WES and CNV-seq are complimentary to each other and be combined in research and clinical diagnosis to improve clinical efficiency and diagnostic yield of multisystem anomalies (Chen et al., 2022).

There are a few limitations in our study. There are certain technical drawbacks for CNV calling based on WES as aforementioned. Small sample size is another major limitation of our study. To optimize study power and increase the possibility of identifying genetic components of HV, we adopted a family-based approach (Borecki and Province, 2008) focusing on familial Hallux Valgus. To find the potentially pathogenic CNVs related to HV, we also filtered the case-only CNVs against all the CNVs carried by healthy individuals in the DGV database. Larger sample size will be needed to further investigate the substantial contribution of various CNV regions in the human genome (Zarrei et al., 2015) to the etiology of HV, and particularly will gain statistical evidence for CNVs of uncertain clinical significance.

In summary, from an unbiased genomic approach, this first study on the structural variants in familial HV gained us new insights into the HV pathogenesis, mediated by genetic variation of immunity and inflammation, and abnormal cytochrome P450 related metabolism. It provides evidence supporting the dominant inheritance mode of HV. Our results suggest that HV is a degenerative joint disease involving the dysregulation of immune system and metabolism system.

## Data Availability

The original contributions presented in the study are publicly available. This data can be found here: https://db.cngb.org/ [Accession number CNP0004036].
