# Supplementary material for: Identification of copy number variants contributing to hallux valgus
Source: Front Genet. 2023 Mar 23;14:1116284. doi: 10.3389/fgene.2023.1116284 (PMC10076598; doi:10.3389/fgene.2023.1116284)
Supplement: Supplementary file 1 [file Table1.pdf]

Supplementary Table 1. The primary clinical characteristics of all the participants in the study.

Family	ID	Kinship	Gender	Current age	Hallux valgus	Degree	Age of onset (self-report)	Surgery	Other conditions (self-report)	BMI	Wearing unfit shoes
F1	I-1	father	male	64	√	mild	45	×	High blood pressure	26.67	√
-	I-2	mother	female	64	×	\	\	×	Heart disease & diabetes	27.1	×
-	II-1	proband	female	29	√	moderate	5	√	\	25.15	√
-	II-2	sister	female	37	√	moderate	10	×	\	30.11	√
F2	I-1	proband	female	64	√	moderate	30	√	High blood pressure	22.32	×
-	I-2	husband	male	65	√	mild	40	×	High blood pressure	25.01	×
-	II-1	daughter	female	38	√	moderate	18	×	\	20.7	×
-	II-2	son	male	31	√	mild	25	×	\	22.89	×
F3	I-1	wife	female	48	×	\	\	×	\	25.39	×
-	I-2	proband	male	45	√	moderate	congenital	√	\	27.68	×
-	II-1	daughter	female	23	√	moderate	congenital	×	\	24.22	×
F4	I-1	mother	female	68	√	moderate	50	√	Gastric carcinoma	25.39	√
-	I-2	father	male	70	√	\	\	×	High blood pressure	24.22	×
-	I-3	aunt	female	58	√	moderate	50	√	\	20.43	√
-	II-1	proband	female	42	√	mild	30	×	\	20.93	√
F5	I-1	mother	female	50	×	\	\	×	\	27.14	×
-	I-2	father	male	54	√	moderate	10	×	\	23.53	×
-	II-1	proband	female	26	√	moderate	10	√	Fourth metatarsal	20.7	×

									short deformity		
Sporadic	S-1	\	female	54	√	moderate	10	√	\	28.04	√
-	S-2	\	female	74	√	severe	50	√	\	22.06	×

Families F1, F3 and F5 were included in our previous study of single nucleotide variants (Jia et al., 2021).

Supplementary Table 2. Specific information for CNVs which passed quality control filtering.

SAMPLE	CNV	INTERVAL	KB	CHR	Q_EXACT	Q_SOME	Q_NON_DIPLOID	Q_START	Q_STOP	MEAN_RD	MEAN_ORIG_RD	GENE
F4-I-3	DUP	1:104120305-104166912	46.61	1	61	90	91	6	57	4.22	175.81	AMY2A,AMY2B
F1-I-2	DEL	1:104160047-104166912	6.87	1	26	99	99	5	26	-6.97	55.54	AMY2A
F1-II-2,F3-I-2	DUP	1:110231214-110235954	4.74	1	40	69	69	12	40	5.46	79	GSTM1
F3-I-2	DUP	1:12853321-12921297	67.98	1	25	99	99	39	23	2.93	78.3	HNRNPCL1,HNRNPCL3,HNRNPCL4,PRAMEF1,PRAMEF11,PRAMEF2
F5-I-1,F4-I-3	DEL	1:12853321-13001478	148.16	1	25	99	99	20	38	-3.46	48.46	HNRNPCL1,HNRNPCL3,HNRNPCL4,PRAMEF1,PRAMEF11,PRAMEF2,PRAMEF4,PRAMEF6,PRAMEF7,PRAMEF8,PRAMEF9
F2-I-2	DEL	1:12884798-13052811	168.01	1	23	99	99	13	46	-3.18	53.07	HNRNPCL1,HNRNPCL3,HNRNPCL4,PRAMEF11,PRAMEF2,PRAMEF4,PRAMEF6,PRAMEF7,PRAMEF8,PRAMEF9
F3-I-2	DUP	1:12979670-12980264	0.59	1	35	35	35	35	20	6.11	123.73	PRAMEF7,PRAMEF8
F1-II-1	DUP	1:13000830-13052811	51.98	1	23	80	80	23	0	5.78	140.31	PRAMEF6,PRAMEF9
F1-II-1	DUP	1:13182983-13414486	231.5	1	28	35	35	0	28	3.27	93.47	HNRNPCL2,PRAMEF10,PRAMEF22,PRAMEF5,PRAMEF6,PRAMEF7,PRAMEF8
F4-I-3	DUP	1:144619833-144814727	194.9	1	28	99	99	10	3	6.29	137.73	NBPF20,NBPF8,NBPF9
F2-I-1	DEL	1:144619833-144816694	196.86	1	22	43	43	20	4	-2.63	84.39	NBPF20,NBPF8,NBPF9
S-2	DEL	1:145273291-145290503	17.21	1	38	38	38	12	18	-3.47	164.27	NBPF20,NBPF25P,NBPF9,NOTCH2NL
F2-I-2	DEL	1:145289722-14536	79.63	1	22	99	99	21	8	-2.59	131.78	NBPF10,NBPF20,NBPF25P,NBPF9

		9352										
F4-II-1	DUP	1:145290394-145299962	9.57	1	72	99	99	27	19	5.26	180.65	NBPF10,NBPF20,NBPF25P,NBPF9
F3-I-1	DUP	1:145296269-145302654	6.39	1	40	81	81	1	13	4.21	133.27	NBPF10,NBPF20,NBPF25P,NBPF9
F5-I-2	DUP	1:145302555-145304047	1.49	1	32	33	33	29	7	4.61	108.7	NBPF10,NBPF20,NBPF25P,NBPF9
S-2	DEL	1:145302555-145304674	2.12	1	52	99	99	15	5	-4.62	125.25	NBPF10,NBPF20,NBPF25P,NBPF9
F5-I-2	DEL	1:146399481-146467777	68.3	1	47	99	99	7	12	-4.3	23.42	NBPF10,NBPF12,NBPF20,NBPF25P
F4-I-3,S-1	DUP	1:148017405-148025907	8.5	1	83	99	99	36	29	6.63	213.54	NBPF19,NBPF26
F5-I-2	DUP	1:148342372-148346952	4.58	1	29	90	90	32	29	4.82	155.29	NBPF14,NBPF25P,NBPF9
F5-I-2	DEL	1:148951238-149376970	425.73	1	32	99	99	11	3	-3.06	41.92	FCGR1C,NBPF25P
F2-I-1	DEL	1:152276739-152278161	1.42	1	22	32	32	19	30	-4.6	18.46	FLG
F2-I-1	DUP	1:161569416-161570083	0.67	1	32	32	32	31	12	4.59	99.38	FCGR2C
F2-I-1	DUP	1:1633031-1639067	6.04	1	35	81	81	21	20	4.78	79.68	CDK11A,CDK11B,MMP23A
F1-I-1	DEL	1:1633031-1669955	36.92	1	23	99	99	21	17	-2.97	20.37	CDK11A,CDK11B,MMP23A,SLC35E2
F5-I-1	DUP	1:1634873-1639067	4.2	1	21	34	34	23	13	4.65	85.91	CDK11A,CDK11B
F5-I-2	DEL	1:1647752-1669955	22.2	1	25	81	81	5	20	-2.97	31.83	CDK11A,CDK11B,SLC35E2
F3-I-1	DUP	1:16911973-172002	288.3	1	46	99	99	9	32	3.49	139.61	CROCCP2,ESPNP,MIR3675,MST1L,MST1P2,NBPF1

		68										
F4-I-1	DEL	1:16915549-17034230	118.68	1	25	99	99	16	0	-4.24	78.77	CROCCP2,ESPNP,MIR3675,MST1P2,NBPF1
F2-I-2	DUP	1:16954955-17034230	79.28	1	29	78	78	43	24	3.67	159.76	CROCCP2,ESPNP,MIR3675,MST1P2
F1-I-2	DEL	1:16954955-17090997	136.04	1	44	99	99	45	3	-4.84	79.34	CROCCP2,ESPNP,MIR3675,MST1L,MST1P2
F4-I-1	DUP	1:17084715-17200268	115.55	1	20	48	48	0	1	3.73	168.19	MIR3675,MST1L
F1-I-2	DUP	1:17215758-17275489	59.73	1	29	99	99	3	11	5.07	108.69	CROCC
F4-I-3	DUP	1:17558-137769	120.21	1	82	99	99	26	16	5.95	123.74	FAM138A,FAM138F,LINC01002,MIR1302-10,MIR1302-11,MIR1302-2,MIR1302-9,OR4F5,WASH7P
F5-II-1,F2-I-2	DUP	1:206137275-206137541	0.27	1	42	42	42	35	15	6.5	248.94	FAM72A
F2-I-2	DEL	1:206566032-206581005	14.97	1	27	35	35	19	10	-3.9	127.03	SRGAP2,SRGAP2D
F3-II-1	DEL	1:220439507-220441094	1.59	1	47	99	99	3	4	-5.19	53.5	AURKAPS1,RAB3GAP2
F3-I-1	DEL	1:22328135-22339006	10.87	1	29	99	99	29	25	-6.6	52.37	CELA3A
F4-II-1	DUP	1:235017344-235017443	0.1	1	30	30	30	28	13	10	309.02	NOT_FOUND
F4-I-3	DUP	1:243219604-243220555	0.95	1	43	99	99	37	23	6.91	245.3	LINC01347
F5-I-2	DEL	1:248756118-248814216	58.1	1	33	76	76	27	11	-4.04	25.48	OR2T10,OR2T11,OR2T27,OR2T35
F1-II-2	DEL	1:2559422	61.42	1	20	99	99	20	7	-5.1	59.33	RHD

		4-25655640										
S-2	DUP	1:40229313-40236252	6.94	1	35	97	98	11	19	4.4	88.92	BMP8B,OXCT2,PPIE
F4-I-2,F4-II-1	DEL	1:40235309-40236252	0.94	1	41	66	66	23	4	-7.35	85.59	BMP8B,OXCT2
F2-I-2	DUP	1:47716497-47728797	12.3	1	34	93	93	12	25	5	113.73	STIL
F4-I-2	DEL	1:87099959-87101828	1.87	1	29	39	39	17	32	-4.78	40.06	CLCA3P
S-2	DEL	1:89476566-89477529	0.96	1	39	43	43	28	6	-6.5	35.32	GBP3
F4-I-3	DUP	10:38734494-38738109	3.62	10	24	87	89	14	19	3.81	167.81	LINC00999
F4-I-1	DUP	10:46751396-46762940	11.54	10	39	46	46	24	27	6.42	65.44	BMS1P5
S-2	DEL	10:47379719-47417050	37.33	10	48	92	99	30	16	-4.57	103.54	FAM35DP
F1-I-1	DEL	10:47409851-47417050	7.2	10	37	40	40	19	6	-6.36	78.63	FAM35DP
S-1	DEL	10:48941148-48952657	11.51	10	32	32	32	3	23	-3.75	7.87	BMS1P5,GLUD1P7
F4-I-3	DUP	10:49239394-49381178	141.78	10	61	92	99	30	5	4.72	135.27	CTGLF12P,FRMPD2
S-2	DEL	10:51469886-51483186	13.3	10	34	35	35	3	12	-4.6	58.48	TIMM23B
F3-II-1	DUP	10:51748651-51768494	19.84	10	33	57	57	21	24	2.78	137.83	AGAP6

F1-II-1	DUP	10:81470965-81587429	116.47	10	57	92	99	11	17	4.53	109.24	NUTM2B,NUTM2B-AS1
F4-I-3	DUP	11:129835-131076	1.24	11	82	92	99	11	50	8.71	94.86	LINC01001
F3-I-1	DUP	11:60974929-60978925	4	11	30	30	30	7	9	5.79	104.77	PGA3
F2-I-2,F3-I-2	DEL	11:67559266-67572847	13.58	11	92	92	99	17	38	-9.18	89.59	FAM86C2P
F3-I-1	DUP	11:89581356-89609208	27.85	11	23	92	99	23	23	3.4	73.1	TRIM53AP,TRIM64B
F4-I-3	DEL	11:89603786-89609208	5.42	11	49	92	99	8	0	-4.5	42.27	TRIM64B
F3-II-1	DUP	11:89608766-89703691	94.93	11	64	67	67	26	47	7.67	83.96	TRIM49D1,TRIM49D2P,TRIM64,TRIM64B
F5-I-1,F4-I-3	DUP	11:89703592-89705409	1.82	11	50	65	65	0	20	5.36	65.49	TRIM64
F1-II-1	DUP	12:10584673-10588591	3.92	12	38	39	39	8	31	4.72	90.59	KLRC2
F5-I-2	DEL	12:10584673-10599337	14.66	12	26	51	51	6	26	-3.67	47.31	KLRC1,KLRC2
S-1	DEL	12:40874947-40876492	1.55	12	22	42	42	14	22	-4.82	55.97	MUC19
F1-I-1	DEL	12:52908403-52914115	5.71	12	36	93	99	24	14	-3.04	50.73	KRT5
F4-I-2	DUP	12:8022413-8088678	66.27	12	32	92	99	26	26	3.07	72.86	SLC2A14,SLC2A3
F4-I-2	DUP	12:8328443-8330116	1.67	12	33	33	33	4	16	4.57	228.45	ZNF705A

F4-II-1	DUP	12:8328443-8333388	4.95	12	22	73	73	10	13	3.98	165.9	FAM66C,ZNF705A
F2-I-2	DEL	12:8329610-8330116	0.51	12	31	32	32	3	31	-5.96	155.15	ZNF705A
F5-I-2,S-2	DEL	12:8383642-8388631	4.99	12	92	92	99	7	38	-9.79	138.2	FAM86FP
F3-I-2,F3-II-1	DEL	12:8391287-8395544	4.26	12	74	92	99	39	36	-6.85	68.45	FAM86FP
F4-I-1	DUP	12:9447376-9456824	9.45	12	21	58	58	39	11	2.8	93.92	NOT_FOUND
F4-I-3	DUP	12:9590581-9600641	10.06	12	44	52	52	20	5	4.21	95.64	DDX12P
F2-I-1	DUP	13:21734029-21742579	8.55	13	40	82	82	6	24	5.85	162.04	SKA3
F5-II-1,F5-I-2	DEL	13:53103360-53106879	3.52	13	21	44	44	20	25	-3.98	49.96	TPTE2P3
F5-II-1	DUP	14:106106483-106139177	32.7	14	21	52	52	7	21	4.11	119.41	ELK2AP,MIR8071-1,MIR8071-2
F1-I-2	DUP	14:19655998-19792919	136.92	14	25	70	70	3	1	4.55	91.94	BMS1P17,BMS1P18,DUXAP10,LINC01296
F2-I-1	DUP	14:19792820-19881346	88.53	14	39	46	46	39	20	4.22	105.96	LINC01296
F3-II-1	DUP	14:19999249-20019742	20.49	14	38	94	99	3	61	7.03	89.16	POTEM
F3-II-1	DUP	15:102495850-102513797	17.95	15	61	82	82	40	6	4.15	88.56	FAM138E,MIR1302-10,MIR1302-11,MIR1302-2,MIR1302-9,MIR6859-1,MIR6859-2,WASH3P
F2-I-2	DUP	15:20636301-20642	6.46	15	25	32	32	8	20	4.52	81.9	HERC2P3

		764										
S-1	DUP	15:20636301-20710794	74.49	15	29	71	71	11	12	3.29	64.86	HERC2P3
S-2	DUP	15:20636301-22137110	1500.81	15	22	94	99	3	6	5.68	100.16	CXADRP2,GOLGA6L6,GOLGA8CP,HERC2P3,LINC01193,MIR3118-2,MIR3118-3,MIR3118-4,MIR5701-1,MIR5701-2,NBEAP1,NF1P2,POTEB,POTEB2,POTEB3
F3-II-1	DEL	15:21932465-22466460	534	15	24	61	61	17	39	-3.04	23.9	CXADRP2,MIR3118-2,MIR3118-3,MIR3118-4,MIR5701-1,MIR5701-2,NF1P2,OR4M2,OR4N3P,OR4N4,POTEB,POTEB2,POTEB3
F3-I-1	DEL	15:22370989-22466460	95.47	15	37	41	41	11	35	-3.98	21.58	OR4N3P,OR4N4
F4-II-1	DUP	15:22709600-22711286	1.69	15	57	69	69	7	8	5.5	108.86	GOLGA8DP
F4-I-1	DUP	15:23260800-23261396	0.6	15	20	83	83	7	11	5.87	144.25	GOLGA8IP
F1-II-1	DEL	15:23308520-23326701	18.18	15	28	94	99	26	27	-4.53	57.62	HERC2P2
F4-I-3	DUP	15:23602418-23613236	10.82	15	22	94	99	16	6	3.06	136.94	GOLGA8S
F4-I-1	DUP	15:23605366-23605957	0.59	15	52	94	99	11	49	6.41	170.39	GOLGA8S
F3-II-1	DUP	15:23605526-23606631	1.11	15	26	83	84	2	3	5.7	201.09	GOLGA8S
F1-II-2,F2-II-2,F3-I-1	DEL	15:24686256-24693149	6.89	15	27	51	51	21	34	-4.11	49.33	PWRN3
F4-I-3	DUP	15:28474600-28479471	4.87	15	22	40	40	4	12	3.47	164.91	HERC2
F5-I-1,F5-I-2	DUP	15:28771330-28776	5.03	15	59	93	98	25	4	4.92	74.66	GOLGA8F,GOLGA8G

		358										
F5-II-1	DUP	15:28771330-28930208	158.88	15	25	77	77	24	15	2.57	92.93	GOLGA8F,GOLGA8G,HERC2P9
F1-II-1	DUP	15:28771330-28947474	176.15	15	40	94	99	29	12	2.71	93.56	GOLGA8F,GOLGA8G,GOLGA8M,HERC2P9
F4-I-2	DEL	15:30376934-30382177	5.24	15	70	92	95	10	9	-6.2	46.07	GOLGA8J
F5-I-1	DEL	15:30865141-30906794	41.65	15	39	94	99	12	18	-3.86	101.6	GOLGA8H,ULK4P1,ULK4P2
F3-I-2	DUP	15:30900531-30903616	3.09	15	23	72	72	16	4	3.95	158.99	GOLGA8H
F4-II-1	DEL	15:32449906-32455593	5.69	15	21	46	46	7	28	-3.24	26.15	CHRNA7
F2-II-1	DEL	15:32512375-32688811	176.44	15	21	43	43	15	21	-4.26	28.11	GOLGA8K
F4-I-3	DUP	15:32512375-32744956	232.58	15	20	94	99	14	7	2.98	67.79	GOLGA8K,GOLGA8O,ULK4P1,ULK4P2,ULK4P3
F5-I-1	DEL	15:32689681-32815466	125.79	15	39	47	47	39	11	-2.46	20.38	GOLGA8K,GOLGA8O,ULK4P1,ULK4P2,ULK4P3,WHAMMP1
F5-I-2	DEL	15:32812033-32820918	8.89	15	32	34	34	11	6	-4.58	35.13	WHAMMP1
F2-I-1	DEL	15:32868105-32895381	27.28	15	22	77	77	6	4	-4.67	43.43	GOLGA8N
F4-I-3	DUP	15:34672296-34818944	146.65	15	22	94	99	21	12	3.52	126.41	GOLGA8A,GOLGA8B,MIR1233-1,MIR1233-2
F4-II-1	DEL	15:34727574-34818944	91.37	15	20	94	99	17	7	-5.14	39.32	GOLGA8A,GOLGA8B
F4-I-2	DEL	15:347275	111.64	15	23	95	99	23	48	-4.7	35.48	GOLGA8A,GOLGA8B,MIR1233-1,MIR123

		74-34839214										3-2
F3-II-1	DUP	15:34817510-34818944	1.44	15	35	92	95	25	6	5.02	152.67	GOLGA8B
F1-II-1	DEL	15:43888568-43940305	51.74	15	28	95	99	16	4	-2.28	30.09	CATSPER2,CKMT1B,STRC
F1-I-1	DEL	15:43924363-43940305	15.94	15	29	93	97	16	4	-2.96	18.84	CATSPER2
F5-I-1,F5-I-2	DEL	15:43932560-43991033	58.47	15	22	95	99	7	15	-4.02	29.62	CATSPER2,CKMT1A
F4-I-1	DUP	15:82920988-83015445	94.46	15	40	95	99	10	43	3.48	57.47	ADAMTS7P1,GOLGA2P10,GOLGA6L10,GOLGA6L17P,GOLGA6L9
F3-I-2	DUP	15:84908054-84909936	1.88	15	21	37	37	11	18	4.58	129.51	GOLGA6L4
F4-I-2	DEL	16:14916275-14983125	66.85	16	51	96	99	40	9	-4.19	40.63	ABCC6P2,NOMO1
F5-II-1,F2-II-1	DUP	16:15069013-15123992	54.98	16	25	95	99	8	31	3.5	166.57	PDXDC1
F3-I-2	DUP	16:15457442-15463921	6.48	16	27	62	62	26	29	4.35	74.75	NPIPA5
F1-I-1	DEL	16:16330748-16364064	33.32	16	22	96	99	22	3	-4.09	14.17	NOMO3
F5-II-1	DUP	16:16330748-16687556	356.81	16	24	95	99	3	4	2.95	67.06	MIR3179-1,MIR3179-2,MIR3179-3,MIR3180-1,MIR3180-2,MIR3180-3,MIR3670-1,MIR3670-2,MIR6511A2,MIR6770-2,NOMO3,NPIPA7,NPIPA8,PKD1P1
F1-II-2	DEL	16:18568944-18609625	40.68	16	39	96	99	17	20	-4.9	37.8	ABCC6P1,NOMO2
F1-I-2	DEL	16:18573317-18609	36.31	16	35	96	99	36	37	-3.22	26.67	ABCC6P1,NOMO2

		625										
F2-I-1,F2-II-2	DUP	16:18893474-19089496	196.02	16	21	96	99	63	19	4.07	98.82	COQ7,TMC7,SMG1,TMC7
F3-I-2,F4-I-1	DUP	16:21475020-21566453	91.43	16	20	42	42	25	26	2.98	91.85	MIR3680-1,MIR3680-2,SLC7A5P2,SMG1P3
F1-I-2	DUP	16:21823074-21823803	0.73	16	45	46	46	25	18	6.67	153.13	RRN3P1
F2-I-2	DEL	16:21823074-21855158	32.09	16	26	96	99	6	24	-3.49	64.89	RRN3P1
F1-I-2,F3-I-1	DUP	16:21854720-21855158	0.44	16	46	49	49	16	36	6.5	143.03	NPIPB4
F3-I-2	DUP	16:22444002-22588174	144.17	16	29	96	99	35	19	3.42	94.38	NPIPB5,RRN3P3,SMG1P1
S-2	DEL	16:22491668-22563901	72.23	16	21	90	92	14	26	-3.53	35.93	NPIPB5,SMG1P1
F1-II-2,F4-II-1	DUP	16:28618126-28619946	1.82	16	44	88	89	7	40	5.97	198.8	SULT1A1
F1-I-1	DUP	16:28769307-28777839	8.53	16	33	36	36	32	34	6.08	53.73	NPIPB9
F3-I-1	DUP	16:29436720-29448721	12	16	60	96	99	42	23	6.15	133.35	SMG1P6
F1-I-2	DEL	16:32163769-32198474	34.71	16	24	96	99	21	12	-4	43.45	HERC2P4
F4-I-3	DUP	16:5134717-5140604	5.89	16	31	95	99	4	8	5.84	355.04	ALG1,FAM86A
F3-II-1	DUP	16:5139067-5145539	6.47	16	23	70	70	8	23	3.95	267.58	FAM86A
F1-I-2,F3-I-1,F3-II-1,	DEL	16:557985	10.06	16	37	51	51	9	5	-4.12	22.9	CES1P1

F4-I-2		97-55808 655										
F3-I-1,F3-II-1	DUP	16:558443 87-55862 898	18.51	16	35	96	99	0	34	6.13	105.04	CES1
F3-I-1	DEL	16:701626 74-70192 805	30.13	16	25	96	99	25	15	-3.88	67.27	PDPR
F3-II-1	DEL	16:701642 87-70192 805	28.52	16	42	96	99	18	18	-3.58	74.71	PDPR
F4-I-3	DUP	16:702081 44-70211 428	3.29	16	48	51	51	23	3	4.96	90.55	CLEC18C
F1-II-1	DEL	16:702081 44-70220 459	12.32	16	25	45	45	26	26	-4.09	22.32	CLEC18C
F1-II-1	DEL	16:720885 00-72094 826	6.33	16	40	93	96	22	2	-3.84	39.18	HP
S-1	DUP	16:743722 94-74372 540	0.25	16	36	50	50	6	31	6.76	134.28	NOT_FOUND
F1-I-2	DEL	16:743945 28-74415 483	20.96	16	21	53	53	20	43	-4.19	54.08	NPIPB15
F2-I-2	DUP	16:743945 28-74455 242	60.72	16	25	96	99	2	9	3.4	89.26	CLEC18B
F5-I-1	DUP	16:744444 97-74445 005	0.51	16	32	33	33	7	17	6.12	190	CLEC18B
F2-II-1	DEL	17:156386 46-15669 073	30.43	17	48	97	99	46	27	-4.48	37.32	CDRT15P2,TBC1D26
F3-I-2	DUP	17:166647 36-16735 563	70.83	17	21	97	99	26	18	4.83	112.37	CCDC144A,KRT16P2,USP32P1
F3-I-2	DEL	17:183431 77-18457 410	114.23	17	44	97	99	11	12	-3.7	55.5	CCDC144B,KRT16P1,LGALS9C,USP32P2

F3-II-1	DEL	17:18414575-18457410	42.84	17	24	97	99	23	11	-3.6	42.7	CCDC144B,USP32P2
F5-I-2,F3-I-2	DEL	17:18497978-18498231	0.25	17	26	35	35	22	17	-6.25	63.29	CCDC144B
F1-II-2,F1-I-2,F5-II-1	DUP	17:18511242-18513642	2.4	17	50	97	99	19	36	6.5	151.54	CCDC144B
F1-II-1	DUP	17:20361461-20361728	0.27	17	54	55	55	10	6	6.91	145.42	LGALS9B
S-1	DUP	17:20405531-20406600	1.07	17	21	46	46	4	16	4.9	136.74	KRT16P3
F4-I-3	DUP	17:20769823-20772004	2.18	17	51	64	64	27	7	7.2	307.87	CCDC144NL,CCDC144NL-AS1
F2-II-1	DEL	17:2583420-2583684	0.27	17	31	31	31	11	7	-6.01	211	PAFAH1B1
F2-II-2	DEL	17:34477646-34540345	62.7	17	22	44	44	17	20	-4.06	13.75	CCL3L1,CCL3L3,CCL4L1,CCL4L2
F4-I-1	DUP	17:34522676-34524089	1.41	17	84	94	96	14	50	8.47	96.57	CCL3L1,CCL3L3
F1-I-1,F3-II-1,F4-I-3	DEL	17:34522676-34641911	119.24	17	20	92	94	14	31	-3.1	13.3	CCL3L1,CCL3L3,CCL4L1,CCL4L2,TBC1D3F,TBC1D3H
F1-II-1	DEL	17:34539688-34625759	86.07	17	26	37	37	20	6	-3.16	9.24	CCL3L1,CCL3L3,CCL4L1,CCL4L2,TBC1D3F,TBC1D3H
F4-I-3	DUP	17:36358293-36398315	40.02	17	40	40	40	8	21	6.38	126.63	NOT_FOUND
F4-I-2,F4-I-3	DUP	17:43590504-43597979	7.48	17	35	79	79	33	32	4.63	133.75	LRRC37A4P
F3-I-1	DEL	17:43591230-43597	6.75	17	37	42	42	10	35	-4.68	73.2	LRRC37A4P

		979										
F4-I-1	DUP	17:44382856-44408872	26.02	17	34	97	99	28	6	5.42	67.3	ARL17A,ARL17B,LRRC37A
F1-I-2	DUP	17:44399669-44408872	9.2	17	38	40	40	3	4	4.68	85.93	ARL17A,ARL17B,LRRC37A
F4-I-1	DUP	17:44415017-44627970	212.95	17	37	97	99	6	16	4.69	48.5	ARL17A,ARL17B,LRRC37A,LRRC37A2,NSFP1
S-2	DUP	17:44594709-44627970	33.26	17	42	44	44	8	43	3.48	86.35	ARL17A,LRRC37A2
F5-I-1	DEL	17:60342062-60345143	3.08	17	36	41	41	34	5	-4.78	65.75	TBC1D3P2
F5-I-2	DUP	18:14183609-14184146	0.54	18	75	80	80	35	49	7.91	298.67	ANKRD20A5P
F2-II-1,F4-I-1	DEL	18:44542994-44549037	6.04	18	40	40	40	21	36	-6.39	2.3	KATNAL2,TCEB3C,TCEB3CL,TCEB3CL2
S-1	DUP	18:77905806-77960852	55.05	18	22	97	99	6	10	3.59	75.91	PARD6G,PARD6G-AS1
F5-I-2	DEL	19:40376313-40384200	7.89	19	35	70	70	15	34	-5.51	22.24	FCGBP
F2-I-2,F4-I-1,S-1	DEL	19:41349692-41356373	6.68	19	34	99	99	20	19	-4.62	55.78	CYP2A6
F2-II-2	DEL	19:41349692-41381820	32.13	19	21	99	99	17	14	-4.44	55.87	CYP2A6,CYP2A7
F3-I-2	DUP	19:43233920-43268464	34.55	19	20	76	76	3	24	2.8	243.59	PSG3,PSG8
F1-I-2	DEL	19:43268068-43522948	254.88	19	58	99	99	26	29	-4.63	44.9	PSG1,PSG10P,PSG11,PSG6,PSG7,PSG8
F2-II-2	DEL	19:435850	1.71	19	30	30	30	24	26	-5.87	236.62	PSG2

		33-43586744										
F2-I-2	DEL	19:43679362-43773606	94.25	19	32	99	99	18	17	-2.68	116.58	PSG4,PSG5,PSG9
F1-I-1,F1-II-2,F4-I-2	DUP	19:43715936-43752836	36.9	19	22	99	99	40	22	4.76	36.72	NOT_FOUND
F2-II-2,F3-I-2	DEL	19:43715936-43773606	57.67	19	35	86	86	13	29	-2.76	68.63	PSG9
F3-I-2	DEL	19:46623582-46627949	4.37	19	23	99	99	23	29	-5.45	0	IGFL3
F3-I-2	DEL	19:49376491-49379264	2.77	19	36	98	99	11	30	-6.37	54.41	PPP1R15A
F4-I-3	DUP	19:50463387-50474650	11.26	19	25	50	50	25	16	6.85	148.89	SIGLEC11
F3-I-1	DUP	19:52146575-52149355	2.78	19	33	51	51	13	26	4.1	45.09	SIGLEC14
F1-II-2,F3-I-2	DEL	19:54744265-54746787	2.52	19	42	99	99	4	12	-4.3	56	LILRA6,LILRB3
F1-I-2	DEL	19:55105667-55106913	1.25	19	26	43	43	20	21	-4.06	167.95	LILRA1
F1-I-2,F4-I-1	DEL	19:55340769-55362002	21.23	19	27	42	42	29	30	-4.05	71.15	KIR2DS4,KIR3DL1,KIR3DL2
F3-I-2	DUP	19:8167567-8370109	202.54	19	23	99	99	7	14	4.15	117.39	CD320,CERS4,FBN3
F3-I-1	DEL	2:107040493-107041728	1.24	2	32	35	35	27	32	-4.67	146.9	RGPD3
F4-I-3	DUP	2:109115994-109116271	0.28	2	67	78	78	12	20	7.81	230.82	GCC2

F3-II-1	DUP	2:109347226-109384914	37.69	2	20	54	54	20	13	2.01	152.43	RANBP2
F3-I-2	DUP	2:109367641-109384914	17.27	2	20	98	99	5	15	2.6	180.3	RANBP2
F5-I-1	DUP	2:110721032-110726193	5.16	2	84	97	99	19	63	8.67	110.65	LIMS3-
S-1	DEL	2:113127657-113138408	10.75	2	41	41	41	18	40	-6.44	37.24	RGPD5,RGPD8
F1-II-2,F4-I-2	DEL	2:114251314-114252893	1.58	2	31	31	31	6	25	-5.86	0	CBWD2,ZNG1B
F1-I-2	DEL	2:114335705-114356263	20.56	2	41	98	99	20	5	-3.37	100.77	FAM138B,MIR1302-3,WASH2P
F4-I-3	DEL	2:114346054-114346355	0.3	2	34	48	48	26	30	-6.66	96.16	WASH2P
F5-I-1	DEL	2:114357222-114358488	1.27	2	46	47	47	25	13	-4.98	196.7	DDX11L2
F1-II-1,F2-II-1	DUP	2:130832128-130869728	37.6	2	43	56	56	3	26	2.91	137.19	POTEF
S-2	DEL	2:131254078-131266493	12.42	2	40	52	52	32	10	-5.13	40.89	POTEI
F3-I-2	DUP	2:131981075-131995908	14.83	2	27	96	99	10	11	2.98	126.72	POTEE
F1-I-2	DEL	2:131985738-132010103	24.37	2	38	45	45	3	5	-3.21	60.36	POTEE
F4-I-1	DUP	2:152435786-152464169	28.38	2	63	70	70	10	31	4.59	56.65	NEB
F5-II-1	DEL	2:179296194-17931	18.99	2	20	98	99	22	17	-3.04	90.87	MIR548N,PRKRA

		5185										
F1-II-2	DUP	2:242690616-242716440	25.82	2	23	56	56	8	39	4.24	127.17	D2HGDH,GAL3ST2
F1-I-1,F1-II-2,F1-II-1	DEL	2:73868064-73912793	44.73	2	37	98	99	16	26	-5.06	25.97	ALMS1P,NAT8
F5-I-1	DEL	2:87754980-87821119	66.14	2	40	61	61	28	16	-4.35	42.56	LINC00152
F5-I-2	DEL	2:96517434-96521962	4.53	2	44	62	62	15	9	-4.41	209.47	ANKRD36C
F3-II-1	DUP	2:97860580-97950783	90.2	2	30	98	99	4	0	3.71	55	ANKRD36,APPAT
F3-II-1,F4-I-3	DEL	2:98088490-98162138	73.65	2	34	77	77	0	19	-3.16	28.67	ANKRD36B
F4-I-3	DEL	20:14864855-14910150	45.3	20	21	82	82	28	17	-4.75	35.14	MACROD2,MACROD2-AS1
F3-I-2	DEL	20:1569188-1592039	22.85	20	37	99	99	19	12	-4.77	0	SIRPB1
F1-II-1,S-2	DEL	20:25751988-25755995	4.01	20	43	99	99	15	15	-6.43	123.67	FAM182B
F4-I-3	DUP	20:25751988-25829625	77.64	20	50	99	99	4	29	4.6	167.96	FAM182B
F1-II-1,S-2	DUP	20:26061957-26131245	69.29	20	22	93	94	25	21	3.86	133.92	FAM182A,NCOR1P1
F2-I-1	DEL	20:34169948-34173270	3.32	20	23	51	51	26	23	-3.3	34.49	FER1L4
F5-I-2	DUP	21:10817296-10817395	0.1	21	30	30	30	28	17	10	254.46	NOT_FOUND
F1-I-2	DEL	21:108172	127.56	21	25	99	99	22	35	-5.14	103.5	TPTE

		96-10944859										
F5-I-1	DEL	21:10906857-10952987	46.13	21	29	99	99	6	29	-3.6	107.36	TPTE
F5-I-2	DEL	21:10951240-11049651	98.41	21	27	57	57	11	7	-2.97	132.28	BAGE2,BAGE3,BAGE4,BAGE5,TPTE
F1-I-1	DEL	21:11049542-11097726	48.19	21	26	38	38	35	26	-4.81	126.18	BAGE,BAGE2,BAGE3,BAGE4,BAGE5
F2-I-2	DEL	21:11097542-11098775	1.23	21	35	35	35	16	18	-6.05	232.48	BAGE,BAGE2,BAGE3,BAGE4,BAGE5
F5-II-1,F5-I-2	DUP	21:38844917-38884868	39.95	21	41	99	99	9	3	4.7	155.07	DYRK1A
F5-I-2	DEL	22:16190779-16231292	40.51	22	20	40	40	10	20	-3.48	37.04	DUXAP8
F3-I-1,F3-II-1	DUP	22:16190779-16449828	259.05	22	51	99	99	27	20	5.41	99.92	DUXAP8,OR11H1,POTEH,POTEH-AS1
F1-I-2	DUP	22:16199664-16231292	31.63	22	35	51	51	28	20	4.16	80.29	PSLNR
F1-II-1	DUP	22:18660780-18838200	177.42	22	22	99	99	21	61	4.01	71.16	GGT3P
F1-II-2	DEL	22:18745348-18898619	153.27	22	26	49	49	13	6	-2.48	52.21	DGCR6,GGT3P
F4-I-3	DUP	22:18837730-18861192	23.46	22	21	85	85	4	30	5.87	90.42	POM121L15P,FAM230F
F4-I-1	DUP	22:20336925-20350514	13.59	22	45	99	99	44	0	4.73	54.38	FAM230A,FAM230G
F1-II-1	DUP	22:20336925-20398764	61.84	22	66	99	99	57	9	4.55	56.41	PI4KAP1,TMEM191B

S-1	DUP	22:20336925-20653856	316.93	22	22	99	99	31	22	5.35	64.21	PI4KAP1,RIMBP3,TMEM191B
F2-I-2	DUP	22:20378388-20461326	82.94	22	29	99	99	25	25	4.31	58.85	PI4KAP1,RIMBP3,TMEM191B
F1-I-1	DEL	22:21063571-21067127	3.56	22	67	99	99	28	11	-5.36	58.53	PI4KA
F1-II-2	DEL	22:21532637-21540843	8.21	22	74	96	97	8	52	-4.27	19.53	FAM230B
F4-I-3	DUP	22:21637691-21662556	24.87	22	22	99	99	18	25	3.26	88.15	POM121L8P
F1-I-1,F5-II-1	DUP	22:21828873-21832432	3.56	22	26	65	65	26	12	5.44	69.71	PI4KAP2
F1-II-1	DUP	22:21838259-21846432	8.17	22	32	49	49	15	26	3.55	80.88	PI4KAP2
F2-I-1	DEL	22:22981588-22989501	7.91	22	28	99	99	32	13	-4.22	74.57	GGTLC2,POM121L1P
F2-I-2	DUP	22:24345642-24400304	54.66	22	24	99	99	12	23	3.68	62.56	GSTT1,GSTTP1,GSTTP2
F1-I-2	DEL	22:25023367-25024076	0.71	22	20	36	36	5	18	-4.65	130.19	GGT1
F1-II-1	DUP	22:25043186-25044144	0.96	22	31	53	53	11	11	6.92	222.88	BCRP3,POM121L10P
F2-I-2	DUP	22:25670895-25857679	186.78	22	28	99	99	27	14	4.15	137.94	CRYBB2P1,IGLL3P,LRP5L,MIR6817
F1-I-1,F1-II-1	DUP	22:38965246-38967405	2.16	22	33	99	99	9	9	5.38	80.58	DMC1
F5-II-1,S-2,F4-I-1	DEL	22:39378413-39388	9.82	22	97	99	99	14	20	-7.64	59.14	APOBEC3A_B,APOBEC3B,APOBEC3B-AS1

		228										
F1-I-1,F1-II-2,F1-II-1, F5-II-1	DEL	22:42522498-42536739	14.24	22	33	99	99	27	31	-6.42	32.87	CYP2D6,CYP2D7,
F4-I-3	DUP	3:125643734-125647875	4.14	3	32	32	32	31	26	3.85	294.88	FAM86JP
F1-II-1	DUP	3:162471514-162621813	150.3	3	29	73	73	20	29	5.71	112.08	NOT_FOUND
S-2	DEL	3:195388615-195389671	1.06	3	38	38	38	37	10	-6.26	11.5	SDHAP2
F5-I-2,F4-I-3	DUP	3:195388615-195400905	12.29	3	22	30	30	4	15	3.29	46.57	SDHAP2
F2-I-1	DUP	3:195416205-195447971	31.77	3	20	96	99	6	16	4.5	143.52	LINC00969,MIR570,MUC20
S-2	DUP	3:195653963-195702788	48.83	3	26	96	99	4	7	4.25	101.03	SDHAP1
F1-I-1	DEL	3:195686911-195692488	5.58	3	29	34	34	15	3	-4.57	78.18	SDHAP1
F1-I-2	DEL	3:195686911-195717122	30.21	3	23	96	99	31	21	-3.93	81.78	SDHAP1
F4-I-3	DEL	3:195698103-195701454	3.35	3	39	39	39	35	6	-6.36	89.38	SDHAP1
F3-I-1	DUP	3:195703919-195709052	5.13	3	34	96	99	30	55	5.68	105.05	SDHAP1
S-2	DUP	3:195708948-195713487	4.54	3	46	92	94	8	47	6.16	110.53	SDHAP1
F2-II-1	DEL	3:197349161-197351080	1.92	3	31	31	31	4	29	-4.5	56.89	NOT_FOUND
F2-I-1	DEL	3:1978945	2.2	3	58	69	69	13	58	-7.26	30.55	FAM157A,FAM157B

		45-19789 6744										
F3-II-1	DUP	3:7547072 6-754800 94	9.37	3	39	97	99	22	3	4.43	293.87	FAM86DP
F5-I-2	DUP	3:7578690 4-757905 28	3.62	3	32	76	76	3	9	4.61	164.12	MIR4273,ZNF717
F1-II-2	DUP	4:1904730 91-19059 9855	126.77	4	37	94	99	15	8	4.3	249.62	LINC01262
F4-I-3	DUP	4:1905810 68-19059 9855	18.79	4	68	94	99	3	27	5.1	239	LINC01262
S-2	DEL	4:1905810 68-19065 1551	70.48	4	22	37	37	10	18	-3.44	142.76	LINC01262
F4-I-3	DUP	4:3943477 -3953010	9.53	4	22	95	99	23	25	4.53	253.37	FAM86EP
F4-I-3	DUP	4:6940333 3-694342 43	30.91	4	61	95	99	6	29	5.81	58.42	UGT2B17
F1-II-1,F4-I-2,F4-II-1	DUP	4:7014621 3-701605 67	14.36	4	34	95	99	4	8	3.72	150.2	UGT2B28
F4-I-3	DEL	4:7014621 3-702100 17	63.8	4	21	95	99	21	17	-4.7	64.65	UGT2B28
F4-I-2	DEL	4:7035933 8-703914 81	32.14	4	32	56	56	12	8	-4.24	42.16	UGT2B4
F5-II-1,F5-I-1	DEL	5:1402225 87-14023 8199	15.61	5	32	94	99	18	6	-4.25	49.71	PCDHA1,PCDHA10,PCDHA2,PCDHA3,PC DHA4,PCDHA5,PCDHA6,PCDHA7,PCDHA 8,PCDHA9
F3-I-2	DUP	5:1404743 74-14062 2423	148.05	5	31	94	99	28	19	4.65	294.93	PCDHB10,PCDHB11,PCDHB12,PCDHB13, PCDHB14,PCDHB16,PCDHB17,PCDHB18, PCDHB19P,PCDHB2,PCDHB3,PCDHB4,PC DHB5,PCDHB6,PCDHB7,PCDHB8,PCDHB 9
F4-I-3	DUP	5:1404743 74-14062	153.15	5	31	94	99	19	22	4.54	264.43	PCDHB10,PCDHB11,PCDHB12,PCDHB13, PCDHB14,PCDHB15,PCDHB16,PCDHB17,

		7522											PCDHB18,PCDHB19P,PCDHB2,PCDHB3,PCDHB4,PCDHB5,PCDHB6,PCDHB7,PCDHB8,PCDHB9
F2-I-2	DUP	5:147653873-147670572	16.7	5	44	62	62	25	31	4.38	102.42		SPINK13
F2-II-2	DUP	5:1572071-1576497	4.43	5	21	41	41	3	19	3.44	167.95		SDHAP3
F5-I-1	DUP	5:177163586-177172031	8.45	5	33	35	35	32	4	3.85	93.86		FAM153A
F1-II-2	DUP	5:177303643-177309688	6.05	5	25	93	99	25	10	5.54	191		NOT_FOUND
F4-I-3	DEL	5:177309315-177311207	1.89	5	24	93	99	35	23	-5.78	72.42		NOT_FOUND
F1-I-2,F5-II-1,F3-II-1,F4-I-2	DUP	5:180375311-180429860	54.55	5	27	93	99	28	6	4.01	65.3		BTNL3,BTNL8
F1-I-1	DUP	5:180376892-180429860	52.97	5	21	33	33	7	23	3.43	45.3		BTNL3,BTNL8
F3-I-2	DEL	5:57667188-57667287	0.1	5	30	30	30	22	14	-10	84.53		NOT_FOUND
F4-II-1	DUP	5:68868225-68878279	10.05	5	31	32	32	20	18	4.42	75.83		GTF2H2C,GTF2H2C_2
F2-I-2	DUP	5:70307097-70308786	1.69	5	23	51	51	23	35	6.68	122.26		NAIP
S-1	DUP	5:70369951-70388915	18.96	5	23	35	35	14	35	4.51	41.79		SNORD13B-2,SNORD13B-1
F1-I-1,F3-II-1,F4-I-3	DUP	5:801196-825377	24.18	5	42	94	99	40	25	5.79	73.63		ZDHHC11
F4-I-3	DUP	5:99716983-99724000	7.02	5	23	31	31	4	15	5.75	185.3		NOT_FOUND
F2-I-2,F3-I-2	DUP	6:1502664	0.26	6	49	52	52	9	43	6.85	209.5		ULBP2

		83-150266744									
F2-II-1,F2-I-1	DEL	6:29855550-29895036	39.49	6	25	93	99	20	9	-3.49	39.58	HCG4B,HLA-H
F1-II-1	DEL	6:29855550-29910411	54.86	6	20	93	99	18	5	-3.34	48.5	HCG4B,HLA-A,HLA-H
F3-I-1,F4-II-1	DEL	6:304590-345946	41.36	6	52	93	99	40	32	-6.43	45.96	DUSP22
F1-II-2	DEL	6:31237703-31239482	1.78	6	20	55	55	31	17	-4.28	104.84	HLA-C
F3-II-1	DUP	6:31237965-31239140	1.18	6	42	43	43	14	23	6.53	155.17	HLA-C
F4-I-1	DUP	6:31323883-31324234	0.35	6	65	79	80	26	13	7.94	143.17	HLA-B
S-1	DEL	6:31994714-31997161	2.45	6	35	56	56	23	5	-5.13	45.69	C4B,C4B_2
F3-II-1	DUP	6:32485468-32522758	37.29	6	22	93	99	22	35	3.88	60.21	HLA-DRB5,HLA-DRB6
F2-I-1	DUP	6:35754737-35765078	10.34	6	28	93	99	22	23	4.62	88.41	CLPS,CLPSL1
S-1	DEL	6:57467044-57512735	45.69	6	22	49	49	2	6	-4.02	106.21	PRIM2
F4-I-1	DEL	7:100333315-100336308	2.99	7	24	71	71	4	13	-3.92	35	ZAN
F3-II-1	DUP	7:100645913-100646189	0.28	7	47	59	59	17	12	7.15	140.33	MUC12
F4-I-1	DUP	7:100645913-100648828	2.92	7	30	61	61	7	15	3.82	108.35	MUC12
F5-I-2	DEL	7:1022462	63.19	7	35	64	64	14	22	-3.48	22.6	POLR2J2,RASA4,RASA4B,SPDYE2,SPDYE

		70-102309461										2B,UPK3BL
F2-I-2	DEL	7:143969519-144068465	98.95	7	23	62	62	6	6	-2.86	49.94	ARHGEF34P,ARHGEF5,OR2A1,OR2A1-AS1,OR2A20P,OR2A42,OR2A9P
F2-II-1	DUP	7:149557462-149577832	20.37	7	65	92	99	5	4	5.25	124.34	ATP6V0E2,ATP6V0E2-AS1,ZNF862
F3-I-2	DEL	7:149557462-149627882	70.42	7	25	92	99	11	5	-3.6	89.05	ATP6V0E2,ATP6V0E2-AS1,ZNF862
F5-II-1	DEL	7:29688574-29690607	2.03	7	63	68	68	9	4	-7.37	92.89	NOT_FOUND
F4-I-1	DEL	7:5939907-5949761	9.86	7	35	92	99	26	25	-4.78	9.77	CCZ1
F1-I-2	DEL	7:5939907-5966188	26.28	7	38	92	99	44	18	-3.59	25.63	CCZ1,RSPH10B,RSPH10B2
F3-I-2	DUP	7:5939907-5968025	28.12	7	22	87	89	17	22	2.6	84.08	CCZ1,RSPH10B,RSPH10B2
S-2	DUP	7:5963430-5983636	20.21	7	53	92	99	37	6	5.34	74.44	CCZ1,RSPH10B,RSPH10B2
F1-II-1,F4-I-3	DUP	7:65150539-65171414	20.88	7	36	38	38	18	16	3.15	90.32	INTS4P2
F5-I-2,F3-I-2	DUP	7:6836192-6844742	8.55	7	36	92	99	25	34	4.77	41.28	CCZ1B,RSPH10B,RSPH10B2
F1-I-2,F4-II-1	DUP	7:6859498-6864391	4.89	7	30	92	99	6	21	4.09	100.71	CCZ1B
F4-I-3	DUP	7:72334375-72337033	2.66	7	40	78	78	26	17	5.78	229.19	SPDYE7P
F3-II-1	DEL	7:72412369-72650022	237.65	7	35	92	99	29	6	-4.13	41.04	GTF2IP1,NCF1B,NSUN5P2,PMS2L2,PMS2P5,POM121,SPDYE8P,STAG3L1,STAG3L3,TRIM74
F3-I-2	DUP	7:72468101-72483824	15.72	7	28	60	60	20	20	5.27	151.33	PMS2L2,PMS2P5,STAG3L1,STAG3L3
F3-I-1	DEL	7:74582328-74653480	71.15	7	28	61	61	18	19	-2.95	27.11	GTF2IP1,NCF1C

F5-I-2	DEL	7:76103117-76126872	23.76	7	23	34	34	3	9	-3.07	32.95	DTX2,FDPSP2
F2-I-2	DEL	7:76641117-76641421	0.3	7	55	66	66	11	16	-7.24	37.15	DTX2P1-UPK3BP1-PMS2P11
F2-II-1,F4-I-1	DEL	7:76641117-76682094	40.98	7	34	51	51	31	32	-3.63	69.57	DTX2P1-UPK3BP1-PMS2P11,PMS2P9
F3-II-1	DEL	7:76668921-76682094	13.17	7	37	92	99	23	32	-6.55	46.55	PMS2P9
F2-II-2	DEL	7:99913339-99930205	16.87	7	25	73	73	24	7	-2.78	151.79	PMS2P1,SPDYE3
F4-I-1	DUP	8:11973465-11979522	6.06	8	83	91	98	37	46	8.6	63.16	FAM66D
F5-I-1	DUP	8:12286133-12408574	122.44	8	45	72	72	8	4	3.32	126.96	FAM86B2
F5-II-1	DEL	8:39238299-39380522	142.22	8	37	92	99	9	23	-5.63	0.16	ADAM3A,ADAM5
F1-I-1	DEL	8:7206582-7680921	474.34	8	22	92	99	6	20	-4.01	29.33	DEFB103A,DEFB103B,DEFB104A,DEFB104B,DEFB105A,DEFB105B,DEFB106A,DEFB106B,DEFB107A,DEFB107B,DEFB4B,FAM66B,FAM90A10P,FAM90A7P,PRR23D1,PRR23D2,SPAG11B,ZNF705G
F2-I-2	DUP	8:7780350-7806846	26.5	8	35	91	98	11	35	6.2	59.82	ZNF705B
F3-I-2	DUP	8:8092000-8094870	2.87	8	34	38	38	7	9	3.45	268.51	FAM86B3P
F1-I-2	DEL	9:107360761-107380314	19.55	9	21	65	65	24	16	-3.52	97.68	OR13C2,OR13C5,OR13C9
F2-I-1	DUP	9:135939788-135947191	7.4	9	66	92	99	4	8	7.32	127.8	CEL
F2-II-1,F2-I-1	DEL	9:141069770-14107	1.29	9	46	84	85	6	15	-4.84	75.86	TUBBP5

		1058										
S-1	DUP	9:141069770-141090772	21	9	90	92	99	21	8	5.56	123.52	TUBBP5
F2-I-1	DEL	9:16048-72875	56.83	9	42	88	90	48	38	-3.54	30.97	FAM138C,MIR1302-10,MIR1302-11,MIR1302-2,MIR1302-9,PGM5P3-AS1,WASH1
F4-I-3	DUP	9:162373-178868	16.5	9	26	50	50	5	20	3.01	114.73	CBWD1
F3-I-1	DUP	9:35440-121619	86.18	9	25	36	36	12	19	2.65	100.05	CBWD1,FAM138C,FOXD4,PGM5P3-AS1
F4-I-3	DUP	9:41237646-41327419	89.77	9	47	82	82	38	46	5.83	79.58	FAM74A1,SPATA31A5,SPATA31A7
F1-II-1,F5-I-1	DUP	9:43614647-43630778	16.13	9	30	92	99	24	42	3.78	96.86	FAM74A7,SPATA31A6
F3-I-2	DUP	9:43849679-43861220	11.54	9	24	32	32	13	29	3.34	88.14	CNTNAP3B,CNTNAP3P2
F1-I-1,F2-I-1,F4-I-1	DUP	9:66466242-66467979	1.74	9	62	92	99	13	10	6.31	111.98	LINC01410
F2-II-2	DEL	9:66466242-67334142	867.9	9	23	92	99	10	28	-2.3	68.1	AQP7P1,LINC01410,PTGER4P2-CDK2AP2P2
F2-II-2	DEL	9:68427740-68433648	5.91	9	20	34	34	10	20	-3.85	162.3	NOT_FOUND
F4-I-3	DUP	9:68427740-68438741	11	9	34	92	99	33	5	5.14	258.03	NOT_FOUND
S-2	DEL	9:68427740-68454391	26.65	9	30	92	99	27	7	-4.68	267.77	NOT_FOUND
F1-I-2	DEL	9:68438467-68438741	0.28	9	31	33	33	7	13	-6.1	188.73	NOT_FOUND
F3-I-2	DUP	9:69200305-69247621	47.32	9	25	92	99	13	6	4.32	100.32	CBWD6,FOXD4L6

F2-II-2,F3-I-2	DUP	9:69382078-69385908	3.83	9	33	50	50	35	9	5.05	130.86	ANKRD20A4
F5-I-2	DUP	9:69424072-69424214	0.14	9	30	30	30	5	24	10	108.17	ANKRD20A4
F3-I-2	DUP	9:99671440-99699699	28.26	9	26	92	99	11	8	3.03	186.07	NUTM2G
F5-I-2	DEL	X:119560319-119564146	3.83	X	27	42	42	11	13	-2.46	41.73	LAMP2
F4-I-3	DUP	X:1255660-1761917	506.26	X	20	99	99	21	23	5.38	152.56	AKAP17A,ASMT,ASMTL,ASMTL-AS1,CRLF2,CSF2RA,IL3RA,MIR3690,P2RY8,SLC25A6
F2-II-1	DUP	X:13328762-13608493	279.73	X	24	99	99	7	7	4.74	99.31	ATXN3L,EGFL6,GS1-600G8.3,LINC01203,MIR6086
F1-II-2	DEL	X:134156136-134186181	30.05	X	30	38	38	2	5	-3.78	213.47	FAM127A,FAM127B,FAM127C
F2-II-1,F2-I-2	DEL	X:134947353-134948203	0.85	X	25	33	33	15	25	-4.51	37.21	CT45A10
F5-I-2,S-2	DEL	X:136653715-136909521	255.81	X	39	72	72	19	15	-3.18	48.57	ZIC3
F3-II-1	DUP	X:140785612-140786564	0.95	X	51	73	73	11	30	5.52	135.35	SPANXC,SPANXD
F5-I-2	DEL	X:145159357-145309456	150.1	X	44	89	89	8	4	-4.95	73.93	NOT_FOUND
F4-I-1	DEL	X:155239478-155257897	18.42	X	21	42	42	4	31	-3.1	14.96	DDX11L16,IL9R
F1-I-1	DUP	X:25583852-25833976	250.12	X	33	41	41	17	31	3.68	61.2	NOT_FOUND
F5-I-2,S-2	DEL	X:25583852-260839	500.09	X	48	99	99	15	40	-4.13	51.45	NOT_FOUND

		38									
F5-II-1,F4-I-2	DUP	X:26629136-26679220	50.09	X	27	40	40	11	5	6.24	245.6	NOT_FOUND
S-2	DUP	X:30377469-30427568	50.1	X	26	45	45	25	3	6.42	169.38	NOT_FOUND
F5-I-2	DEL	X:31137743-31139105	1.36	X	36	72	72	31	2	-3.55	46.1	DMD
S-2	DUP	X:42737477-42987376	249.9	X	42	99	99	6	5	4.36	126.89	NOT_FOUND
S-2	DUP	X:45810951-45910250	99.3	X	43	46	46	14	4	4.89	116.69	NOT_FOUND
F5-I-1,F3-II-1	DEL	X:55102957-55117959	15	X	23	43	43	5	13	-3.47	123.55	PAGE2,PAGE2B
F1-II-2	DEL	X:57935649-58084567	148.92	X	21	36	36	13	23	-2.94	120.36	ZXDA
F5-I-2	DEL	X:65906747-66008896	102.15	X	30	30	30	25	8	-4.53	72.31	NOT_FOUND
F4-I-1	DEL	X:82501942-82602827	100.89	X	41	73	73	23	27	-5.66	57.04	NOT_FOUND
S-2	DEL	Y:14098462-14299354	200.89	Y	36	56	56	10	36	-5.33	0	NOT_FOUND
F5-I-2	DUP	Y:16017066-16218727	201.66	Y	33	33	33	33	31	6.37	85.02	VCY,VCY1B
F3-I-2	DUP	Y:23710674-24050358	339.69	Y	24	99	99	24	49	7.52	138.12	RBMY1A1,RBMY1B,RBMY1D,RBMY1E,TTTY13
F1-I-2	DUP	Y:25375678-25375787	0.11	Y	31	31	31	31	31	10	160.32	DAZ2,DAZ3,DAZ4
F5-I-2,S-2	DUP	Y:3019401	678.13	Y	22	65	65	30	15	3.95	69.69	TGIF2LY

	-3697526

SAMPLE: sample name; CNV: type of copy number variation (DEL or DUP); INTERVAL: location of the CNV; KB: length of the CNV; CHR: chromosome where CNV is located; Q_EXACT: the exact CNV event Phred-scaled quality along the entire interval; Q_SOME: some CNV event Phred-scaled quality in the interval; Q_NON_DIPLOID:not being diploid Phred-scaled quality, i.e., DEL or DUP; Q_START: CNV "left" breakpoint Phred-scaled quality; Q_STOP: CNV "right" breakpoint Phred-scaled quality ; MEAN_RD:CNV mean normalized read depth (z-score); MEAN_ORIG_RD: CNV mean read depth (reads); GENE:gene name.

Supplementary Table 3.Case-only CNV information

SAMPLE	CNV	INTERVAL	KB	CHR	Q_EXACT	Q_SOME	Q_NON_DIPLOID	Q_START	Q_STOP	MEAN_RD	MEAN_ORIG_RD	GENE
F3-I-2,F1-II-2	DEL	1:110231214-110235954	4.74	1	34	56	56	35	31	-5.13	0	GSTM1
S-1,F4-I-3	DEL	1:148017405-148025907	8.5	1	57	79	79	13	29	-5.62	84.53	NBPF19,NBPF26
F2-I-2,F5-II-1	DEL	1:206137275-206137541	0.27	1	83	91	91	32	12	-8.38	101.22	FAM72A
F4-II-1,F4-I-2	DEL	1:40235309-40236252	0.94	1	45	63	63	28	11	-7.29	75.19	BMP8B,OXCT2
F3-I-2,F2-I-2	DUP	11:67559266-67572847	13.58	11	29	92	99	39	29	5.09	205.93	FAM86C2P
F5-I-2,S-2	DEL	12:8383642-8388631	4.99	12	92	92	99	7	38	-9.79	138.2	FAM86FP
F3-II-1,F3-I-2	DEL	12:8391287-8395544	4.26	12	92	92	99	51	26	-7.74	58.75	FAM86FP
F5-I-2,F5-II-1	DEL	13:53103360-53106879	3.52	13	28	93	99	10	27	-5.26	43.93	TPTE2P3
F2-II-2,F1-II-2	DEL	15:24686256-24693149	6.89	15	25	91	94	20	5	-4.9	37.88	PWRN3
F2-II-1,F5-II-1	DEL	16:15069013-15123992	54.98	16	32	96	99	4	12	-4.76	65.34	PDXDC1
F2-I-1,F2-II-2	DUP	16:18893474-19089496	196.02	16	21	96	99	63	19	4.07	98.82	COQ7,TMC7,SMG1
F4-II-1,F1-II-2	DUP	16:28618126-28619946	1.82	16	22	44	44	13	22	4.85	142.21	SULT1A1
F4-I-2,F3-II-1	DUP	16:55798597-55808655	10.06	16	22	70	70	56	3	4.49	85.28	CES1P1
F3-I-2,F5-I-2	DUP	17:18497978-18498231	0.25	17	27	43	43	18	26	6.52	114.45	CCDC144B
F5-II-1,F1-II-2	DEL	17:18511242-18513642	2.4	17	45	65	65	17	40	-5.38	59.85	CCDC144B
F4-I-3,F3-II-1,F1-I-1	DUP	17:34522676-34641911	119.24	17	34	97	99	28	4	3.68	63.22	CCL3L1,CCL3L3,CCL4L1,CCL4L2,TBC1D3F,TBC1D3H
F4-I-3,F4-I-2	DUP	17:43590504-43597979	7.48	17	37	97	99	7	25	5.25	137.87	LRRC37A4P
S-1,F2-I-2	DEL	19:41349692-41356373	6.68	19	33	99	99	25	33	-5.27	39.09	CYP2A6
F4-I-2,F1-II-2,F1-I-1	DEL	19:43715936-43752836	36.9	19	22	99	99	38	16	-4.62	0.09	NOT_FOUND
F3-I-2,F2-II-2	DUP	19:43715936-43773606	57.67	19	32	99	99	26	35	3.41	116.32	PSG9
F3-I-2,F1-II-2	DUP	19:54744265-54746787	2.52	19	24	99	99	21	2	4.55	100.06	LILRA6,LILRB3
F4-I-2,F1-II-2	DUP	2:114251314-114252893	1.58	2	37	37	37	11	33	6.14	78.27	CBWD2,ZNG1B
F2-II-1,F1-II-1	DEL	2:130832128-130869728	37.6	2	42	98	99	16	26	-3.8	66.78	POTEF
F1-II-1,F1-II-2,F1-I-1	DEL	2:73868064-73912793	44.73	2	54	99	99	31	34	-4.7	54.85	ALMS1P,NAT8
F4-I-3,F3-II-1	DEL	2:98088490-98162138	73.65	2	28	64	64	0	16	-3.04	33.88	ANKRD36B
S-2,F1-II-1	DUP	20:25751988-25755995	4.01	20	42	78	78	16	3	5.55	105.71	FAM182B
S-2,F1-II-1	DEL	20:26061957-26131245	69.29	20	47	99	99	32	12	-4.38	85.87	FAM182A,NCOR1P1
F5-I-2,F5-II-1	DUP	21:38844917-38884868	39.95	21	32	99	99	25	9	3.51	90.17	DYRK1A
F5-II-1,F1-I-1	DUP	22:21828873-21832432	3.56	22	42	68	68	27	16	5.52	181.81	PI4KAP2
F1-II-1,F1-I-1	DUP	22:38965246-38967405	2.16	22	31	99	99	7	27	5.76	164.16	DMC1
S-2,F5-II-1	DUP	22:39378413-39388228	9.82	22	30	58	58	5	24	4.31	58.35	APOBEC3A_B,APOBEC3B,APOBEC3B-AS1
F1-II-2,F1-I-1,F1-II-	DEL	22:42522498-42536739	14.24	22	34	90	91	25	33	-6.12	73.77	CYP2D6,CYP2D7

1,F5-II-1												
F4-I-3,F5-I-2	DUP	3:195388615-195400905	12.29	3	31	96	99	34	24	5.71	139.24	SDHAP2
F4-II-1,F4-I-2,F1-II-1	DEL	4:70146213-70160567	14.36	4	53	95	99	12	4	-5.44	50.46	UGT2B28
F3-II-1,F4-I-2,F5-II-1	DEL	5:180375311-180429860	54.55	5	72	93	99	27	16	-6.38	0.75	BTNL3,BTNL8
F3-II-1,F1-I-1	DUP	5:801196-825377	24.18	5	36	94	99	18	23	7.46	108.98	ZDHHC11
F3-I-2,F2-I-2	DEL	6:150266483-150266744	0.26	6	61	63	63	4	28	-7.22	128.45	ULBP2
F2-I-1,F2-II-1	DEL	6:29855550-29895036	39.49	6	34	93	99	17	11	-4.07	35.17	HCG4B,HLA-H
F4-I-3,F1-II-1	DUP	7:65150539-65171414	20.88	7	44	45	45	44	6	3.22	80.65	INTS4P2
F3-I-2,F5-I-2	DEL	7:6836192-6844742	8.55	7	24	43	43	12	20	-2.99	0	CCZ1B,RSPH10B,RSPH10B2
F2-I-1,F2-II-1	DEL	9:141069770-141071058	1.29	9	28	38	38	9	11	-3.93	78.17	TUBBP5
F2-I-1,F1-I-1	DEL	9:66466242-66467979	1.74	9	35	65	65	20	33	-4.4	105.18	LINC01410
F3-I-2,F2-II-2	DEL	9:69382078-69385908	3.83	9	41	67	67	6	18	-5.51	73.99	ANKRD20A4

SAMPLE: sample name; CNV: type of copy number variation (DEL or DUP); INTERVAL: location of the CNV; KB: length of the CNV; CHR: chromosome where CNV is located; Q_EXACT: the exact CNV event Phred-scaled quality along the entire interval; Q_SOME: some CNV event Phred-scaled quality in the interval; Q_NON_DIPLOID:not being diploid Phred-scaled quality, i.e., DEL or DUP; Q_START: CNV "left" breakpoint Phred-scaled quality; Q_STOP: CNV "right" breakpoint Phred-scaled quality ; MEAN_RD:CNV mean normalized read depth (z-score); MEAN_ORIG_RD: CNV mean read depth (reads); GENE:gene name.

Supplementary Table 4.Significantly enriched KEGG pathways.

Term	Database	ID	Input gene	Background gene	P-Value	Corrected P-Value	Input
Drug metabolism - cytochrome P450	KEGG PATHWAY	hsa00982	5	72	1.27E-07	3.28E-06	GSTM1|UGT2B28|CYP2A6|CYP2D6|CYP2D7
Metabolism of xenobiotics by cytochrome P450	KEGG PATHWAY	hsa00980	5	76	1.64E-07	3.28E-06	GSTM1|UGT2B28|CYP2A6|CYP2D6|CYP2D7
Chemical carcinogenesis	KEGG PATHWAY	hsa05204	4	82	9.49E-06	9.93E-05	GSTM1|SULT1A1|CYP2A6|UGT2B28
Salmonella infection	KEGG PATHWAY	hsa05132	4	83	9.93E-06	9.93E-05	CCL4L1|CCL4L2|CCL3L3|CCL3L1
Viral protein interaction with cytokine and cytokine receptor	KEGG PATHWAY	hsa04061	4	100	2.01E-05	1.56E-04	CCL4L1|CCL4L2|CCL3L3|CCL3L1
Toll-like receptor signaling pathway	KEGG PATHWAY	hsa04620	4	104	2.33E-05	1.56E-04	CCL4L1|CCL4L2|CCL3L3|CCL3L1
Cytokine-cytokine receptor interaction	KEGG PATHWAY	hsa04060	5	294	9.56E-05	5.46E-04	CCL4L1|CCL4L2|CCL3L3|CCL3L1|BMP8B
Chemokine signaling pathway	KEGG PATHWAY	hsa04062	4	190	2.26E-04	1.13E-03	CCL4L1|CCL4L2|CCL3L3|CCL3L1
Drug metabolism - other enzymes	KEGG PATHWAY	hsa00983	3	79	2.75E-04	1.22E-03	GSTM1|CYP2A6|UGT2B28
Human cytomegalovirus infection	KEGG PATHWAY	hsa05163	4	225	4.24E-04	1.70E-03	CCL4L1|CCL4L2|CCL3L3|CCL3L1

Supplementary Table 5.Hereditary CNV

SAMPLE	CNV	INTERVAL	KB	CHR	Q_EXACT	Q_SOME	Q_NON_DIPLOID	Q_START	Q_STOP	MEAN_RD	MEAN_ORIG_RD	GENE
F1-I-1,F1-II-1,F1-II-2	DEL	2:73868064-73912793	44.73	2	37	98	99	16	26	-5.06	25.97	ALMS1P,NAT8
F1-I-1,F1-II-1,F1-II-2	DEL	22:42522498-42536739	14.24	22	33	99	99	27	31	-6.42	32.87	CYP2D6,CYP2D7
F2-II-2,F2-I-1	DUP	16:18893474-19089496	196.02	16	21	96	99	63	19	4.07	98.82	COQ7,SMG1,TMC7
F2-II-1,F2-I-1	DEL	6:29855550-29895036	39.49	6	25	93	99	20	9	-3.49	39.58	HCG4B,HLA-H
F2-II-1,F2-I-1	DEL	9:141069770-141071058	1.29	9	46	84	85	6	15	-4.84	75.86	TUBBP5
F2-II-1,F2-I-1	DEL	X:134947353-134948203	0.85	X	25	33	33	15	25	-4.51	37.21	CT45A10
F3-I-2,F3-II-1	DEL	12:8391287-8395544	4.26	12	74	92	99	39	36	-6.85	68.45	FAM86FP
F4-I-2,F4-II-1	DEL	1:40235309-4023625	0.94	1	41	66	66	23	4	-7.35	85.59	BMP8B,OXCT2

		2										
F4-I-2,F4-I-3,F4-II-1	DEL	4:70146213-70160567	14.36	4	64	95	99	15	3	-6.03	58.92	UGT2B28
F5-I-2,F5-II-1	DUP	21:38844917-38884868	39.95	21	41	99	99	9	3	4.7	155.07	DYRK1A
F5-I-2,F5-II-1	DEL	13:53103360-53106879	3.52	13	21	44	44	20	25	-3.98	49.96	TPTE2P3

SAMPLE: sample name; CNV: type of copy number variation (DEL or DUP); INTERVAL: location of the CNV; KB: length of the CNV; CHR: chromosome where CNV is located; Q_EXACT: the exact CNV event Phred-scaled quality along the entire interval; Q_SOME: some CNV event Phred-scaled quality in the interval; Q_NON_DIPLOID:not being diploid Phred-scaled quality, i.e., DEL or DUP; Q_START: CNV "left" breakpoint Phred-scaled quality; Q_STOP: CNV "right" breakpoint Phred-scaled quality ; MEAN_RD:CNV mean normalized read depth (z-score); MEAN_ORIG_RD: CNV mean read depth (reads); GENE:gene name.

Supplementary Table 6.qPCR primers for each CNV and GAPDH

Gene	Genomic(GRCh37)	Product length	Forward primer 5'→3'	Reverse primer 5'→3'
CYP2D6,CYP2D7	chr6:29875143-29875243	88	ACCACTCCTGAACCCCCATTC	GCACCCACACATAATGCAGAGG
HCG4B,HLA-H	chr22:42529419-42529519	101	TGGCCTCTCTCCATAGTCACA	TTCTGGAGTTGTAGGGTAAAATAAATG
GAPDH	chr12:6645165-6645265	101	CACCCGCCCCAGTCTCTG	AACTCAAAGGGCAGGAGTAAAGG
